# A Focused Review on the Flexible Wearable Sensors for Sports: From Kinematics to Physiologies

**DOI:** 10.3390/mi13081356

**Published:** 2022-08-20

**Authors:** Lei Liu, Xuefeng Zhang

**Affiliations:** 1 Department of Sports, Xi’an Polytechnic University, Xi’an 710048, China; 2 Shaanxi Key Laboratory of Nano Materials and Technology, Xi’an University of Architecture and Technology, Xi’an 710055, China; 3 School of Mechanical and Electrical Engineering, Xi’an University of Architecture and Technology, Xi’an 710055, China

**Keywords:** sport monitoring, wearable sensors, flexible sensors, kinematics, physiologies

## Abstract

As an important branch of wearable electronics, highly flexible and wearable sensors are gaining huge attention due to their emerging applications. In recent years, the participation of wearable devices in sports has revolutionized the way to capture the kinematical and physiological status of athletes. This review focuses on the rapid development of flexible and wearable sensor technologies for sports. We identify and discuss the indicators that reveal the performance and physical condition of players. The kinematical indicators are mentioned according to the relevant body parts, and the physiological indicators are classified into vital signs and metabolisms. Additionally, the available wearable devices and their significant applications in monitoring these kinematical and physiological parameters are described with emphasis. The potential challenges and prospects for the future developments of wearable sensors in sports are discussed comprehensively. This review paper will assist both athletic individuals and researchers to have a comprehensive glimpse of the wearable techniques applied in different sports.

## 1. Introduction

The everlasting pursuit of the “Faster, Higher, Stronger” spirit in sports propels the ceaseless advances of exercise and sports sciences. The continuous breakthrough of human beings in competitive sports is not only due to the advanced training concept but also strongly supported by the high-end instruments for sports monitoring, analysis and evaluation. Many commercially available systems, e.g., video motion capture systems, BSXinsigh, Polar sports tester, Keiser fitness equipment, Xsens 3D motion trackers, etc., have been utilized during exercise to detect and analyze the posture and trajectory of the body and measure the physical information of respiration, heart rate, blood oxygen, blood lactate [1]. The obtained results, acting as a golden standard, can provide data support for formulating scientific plans and preventing fatigue or injuries. However, these existing technologies also have certain shortcomings. For example, the video technology cannot quantitatively analyze the force-generating process, and the common physiological monitoring systems often suffer from their poor wearing comfort, low sensitivity and weak capacity in real-time monitoring [2,3].

In the past decade, wearable sensors have taken the predominant role in seamlessly interfacing individual states and monitoring/analyzing systems due to their inherent features (flexibility, ultrathinness, stretchability and lightweight) and high sensing performances (sensitivity, response time and multifunction) [4]. Huge advances have been achieved in developing versatile. flexible and wearable devices to monitor the motion and gesture of the human body, detect the imposed tactile, force and pressure, and measure the parameters of physical conditions [5,6,7,8,9,10,11]. These superiorities contribute a lot to the numerous intriguing practical applications in healthcare, the medical industry, smart homes, internet of things and many other fields of interest. In the meantime, the realization of such promising applications requires sensors that are capable of acquiring abundant physical and biochemical parameters from the human body, such as strain, force, pressure, temperature, amounts and concentrations of metabolites [12,13,14,15,16,17,18]. Motivated by the great potential of wearable sensors, these bendable and flexible devices have also been conformably attached to the body of an athlete and succeeded in detecting a great deal of signals for competitive performances and physical conditions in different sports, such as swimming, running, weightlifting, football, volleyball, racquet sports, fitness actions and so on. 

**Figure 1:**
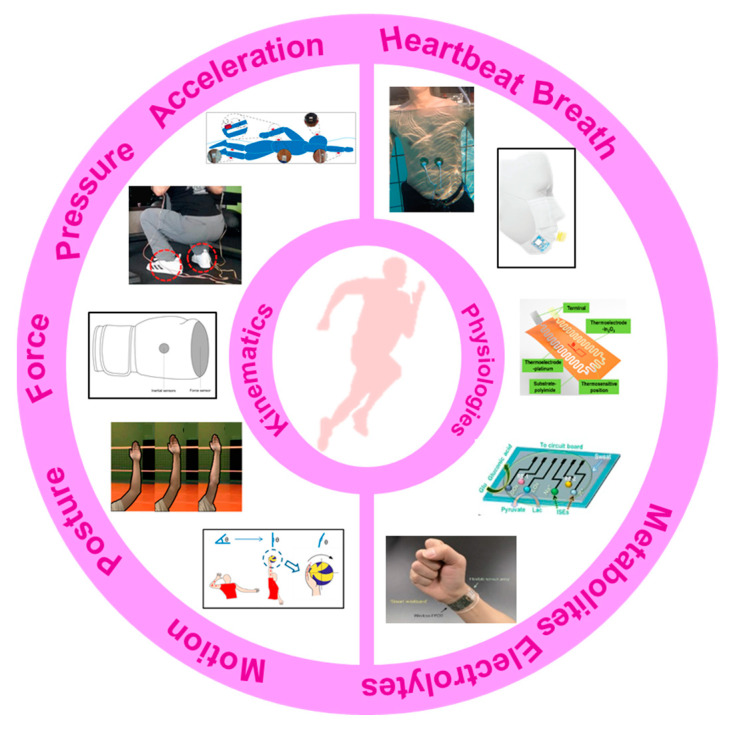
The main concerns of this paper: the monitored indicators and available wearable devices. Reproduced with permissions from [31,32,33,34,35] and under the terms and conditions of the Creative Commons Attribution license of [36,37,38,39].

Many excellent reviews have summarized the progress in flexible, wearable sensors, focusing on emerging materials, fabrications, promotion strategies and applications in healthcare [19,20,21,22,23,24]. Additionally, a few reviewing works put their attention on the wearable system in sports. The importance of accelerating the integration of wearable sensors into recreation and competitive sports is discussed [3]. Wearable sensors used in consumer sports and persons with disabilities are also systematically reviewed [25,26]. The devices and analyzing techniques for several kinematical parameters, e.g., motions [27,28], biomechanics of the upper limbs [29] and shock impacts [30], are summarized. Many of these reviews only focus on one aspect of applying wearable sensors in sports, and the mentioned devices are mainly conventional inertial, force/pressure sensors, which lack technique comprehensiveness and relevance to the latest wearable technology. This review, thus, provides a holistic view of recent developments in flexible, wearable sensors in sports. The main concerns of this paper can be found in Figure 1. A brief introduction of the target indicators, from kinematics to physiologies, is described in Section 2. Commonly used flexible sensors for monitoring kinematical and physiological signals are reviewed in Section 3 and Section 4, with an emphasis on the emerging applications of these devices in monitoring the motions and physical conditions of athletes. In Section 5, multifunctional devices for sports are simply introduced. Then, Section 6 briefly discusses the existing challenges and promising solutions for practical researches. The conclusions are provided in the final section. 

## 2. Indicators in Sports Monitoring

Using systematic, scientific monitoring systems to monitor performance indicators has become a critical issue in modern sports, which can be very helpful in improving the performances of athletes. The obtained results can be used to correct the motion/posture, monitor the real-time response of people and update the training schedules in time. Consequently, the optimized schemes can promote competitiveness, reduce excessive fatigue and avoid unnecessary injuries. Generally, the monitored indicators in sports can be categorized as follows: (1) kinematical indicators, including posture, motion, force and acceleration; (2) physiological indicators, including vital signs (e.g., breath, pulse, ECG, heart beating, blood pressure, temperature, SpO_2_, etc.) and metabolites during and after exercises (e.g., glucose, pH, electrolytes, lactic acid, etc.). The monitoring of many indicators has been realized by using flexible, wearable sensors.

### 2.1. Kinematic Indicators

Kinematic indicators are a series of physical parameters that deal with the postures and motions of objects. Firstly, posture, namely, the deformation of body parts, can characterize the direction, amplitude and frequency of motions. Effective monitoring can help with detecting posture defects, acquiring personal characteristics to meliorate the training strategies and reducing injuries. For example, the movement of lower limbs can show a runner’s stride frequency, step length and the joint angles of the knee/ankle, which is valuable in analyzing, evaluating and then optimizing the strategies of gait, step and stride frequency [40]. Secondly, the measurement of contact forces is also conducted in kinematical monitoring. Plantar force shows the contact between the foot and the ground during the exercise. The obtained results can indicate the gait and arch status of objects and then be used to optimize striding habits and develop tailored shoes [41,42,43,44]. Moreover, the contact status between hands and equipment is also important in many throwing (e.g., shot put) and racket sports (e.g., badminton). Finally, in confrontational sports, such as soccer, basketball and American football, athletes often suffer from shock impacts and proper monitoring can play an important role in improving sport wares and avoiding injury. The following will introduce the indicators involved in exercise monitoring in different body parts. 

#### 2.1.1. Hand and Foot

The hand is the most active part of our body, and its motions play an important role in many sports. The throwing activities require a good combination of hand movements and full-body movements, and the hand shape is also closely related to the hitting quality of volleyball and handball. In addition, the monitoring of grip and exertion is also of great significance for badminton, ping pong and tennis. The foot provides support for human motions and inevitably sustains tremendous pressure for a long time. The variations in the contact area and plantar pressure accurately indicate the gait and forcing process of the lower limb. Thus, measurement and analysis of plantar parameters are crucial in performance monitoring and injury prevention for athletes.

**Figure 2:**
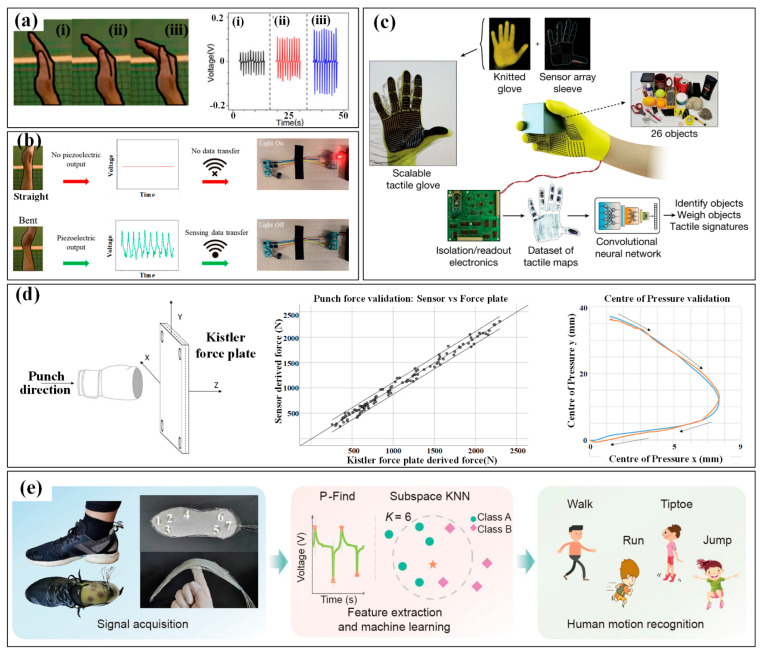
The kinematic indicators in hand and foot: (**a**) the three bending state (i–iii) of hand in volleyball and the corresponding monitoring results; (**b**) the alarming system for hand gesture monitoring [38]; (**c**) tactile maps in grasp generated by the knitted glove [47]; (**d**) the punch forces in boxing measured by a piezoresistive pressure sensing system and verified by a Kistler force plate [37,48]; (**e**) the plantar pressure distribution measured by a printed insole to distinct motions [50]. Reproduced with permissions from Springer Nature (2019) [47]. Reproduced with permissions from Springer Nature (2022) [50].

Hand monitoring mainly involves finger bending and hand contact. Monitoring the bending of the finger/palm can be easily conducted by wearing flexible sensing strips on target fingers, whose movement causes bending/stretching strains in the strips and then induces a variation in their electrical parameters [45]. By carefully designing the sensing mechanism and structural configuration, the finger curvatures can be felicitously converted into electrical variations. However, the directly mounted single strips may induce uncomfortableness to the covered skin, and the suspended electrical wires for power and signals will also become a mess and unreliable if several strips are adhered. Thus, several glove-like devices have been developed to improve the wearability and comfortableness [46]. A larger substrate is utilized to integrate the sensing strips, electrical wires and connectors, which offers an easier and more reliable way to wear the device. With the help of these sensing devices, abundant finger motions have been measured, such as clenching, gesticulating, wheeling a computer mouse and so on. More recently, several self-powered sensors have been proposed to monitor the bending of the finger/palm. An elegant example is a wearable motion sensor for monitoring the spiking gesture of volleyball athletes [38]. Flexible piezoelectric polyvinylidene difluoride (PVDF) film is utilized to convert the mechanical energy of athletes into electricity, generating a voltage signal corresponding to bending angles. As shown in Figure 2a, different angles of the palm are recognized from output voltages. Combining a wirelessly transmitting system and a big sports data platform, the generated signal can be used to real-timely monitor the posture and provide guidance for volleyball players’ daily training (Figure 2b). 

As for hand contact, efficient monitoring can be realized by setting sensitive units in the regions that participate in touching or gripping. Similarly, the units can be arranged in an individual form at various interesting points or integrated into a sensing array to obtain the distribution of tactile forces. The latter scheme is relatively more popular, and many flexible, customizable smart gloves have been designed to obtain tactile information from the entire hand. A typical example is a low-cost, scalable tactile glove that covers the full hand with an array of tactile sensors [47]. As shown in Figure 2c, the 548-unit sensing array is assembled onto the surface of a knitted glove, and used to generate unique tactile maps when grasping different objects such as Coke cans, balls, batteries, erasers and etc. The obtained normal forces of grasping motion fall into the range of 30 mN to 0.5 and can be used to form tactile videos with a frame rate of about 7.3 Hz. Wearable pressure monitoring has also been applied in the measurement and analysis of comprehensive punch parameters in boxing [37,48]. The developed piezoresistive pressure sensing system is integrated into a 12-ounce boxing glove certified by the International Amateur Boxing Association to measure the punch forces, and a Kistler force plate is used for verification (Figure 2d). The results show good consistency both in force and pressure center between the values obtained from the sensing system and force plate. 

The monitoring of plantar pressure has attracted great interest for many years, and several commercialized devices have been taken into markets. Relevant devices include mat devices (e.g., Sports Balance Analyzer™, Footprint Plus™ and Emed^®^-systems, etc.) and in-shoe devices (e.g., F-Scan™ system and Pedar^®^). For example, Amaro et al. used the Pedar^®^ system to evaluate the plantar pressure of players in five different basketball motions [49]. The results showed that no significant statistical differences were found between the two seasons, but a slight decrease was observed throughout the sporting season. These commercialized products feature high resolution, great accuracy, excellent reliability and mature algorithms for performance evaluation, and have been widely used in the evaluation of gait. Meanwhile, the mat devices cannot maintain long-length monitoring for walking or running, and the in-shoe devices often suffer from insufficient integration with professional sneakers. Moreover, the relatively higher cost also prevents this equipment from having large-scale applications in national sports. Thus, the academic researches on wearable plantar pressure measuring systems mainly focuses on improving wearability, integration and comfort, and decreasing the fabrication cost, structural complexity and operative difficulty. Soft materials are made into insoles to realize the monitoring in a wearable, comfortable and integrated way without the high price or complex composition. Corresponding to the desired parameter, the sensing elements, usually force and pressure sensors, can be individually arranged in certain areas of the sole (e.g., great toe, midfoot, metatarsal and heel), or assembled into an array to cover the entire sole. For example, Yang et al. put seven sensing elements into a printed insole to monitor the plantar pressure distribution of the human body and distinct different motions. As shown in Figure 2e, the obtained pressure distributions differ significantly in the motions of walking, running, tiptoeing and jumping [50].

#### 2.1.2. Trunk and Limb

**Figure 3:**
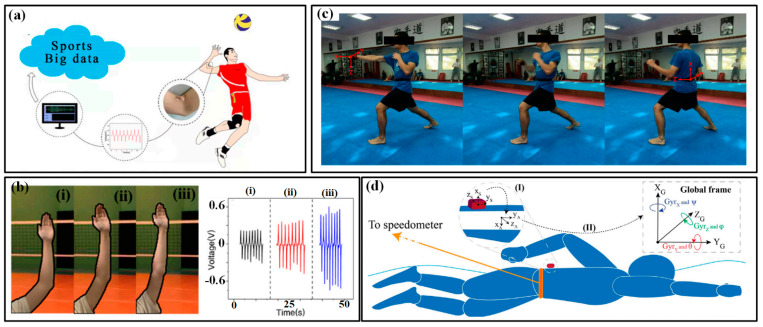
The kinematic indicators in trunk and limb: (**a**) bending angles of the elbow in volleyball and (**b**) monitored results of three bending state of elbow (i–iii) [38]; (**c**) the use of IMUs in karate [58]; (**d**) the use of IMUs in swimming [59].

The motions of the trunk and limbs are the main power sources for various sports, which can provide speed or acceleration for movements and control their magnitudes. The information on the speed/ acceleration and magnitude is a very important domain for evaluating the performances of athletes. The reciprocating bending deformation of the waist, shoulder, elbow, hip and knee undertakes the most work in generating these admired motions. Flexible sensors can be installed at these joints to real-timely monitor the bending and then indicate the motion parameters. Xu et al. mounted a multifunctional epidermal sensor on the lower back of a volunteer to monitor the strain in the waist region when lifting a heavy load. The obtained signals can distinguish the motions of bending-standing and squatting-standing [51]. Combined with synchronous EMG signals, the captured information can be very helpful in preventing excessive muscle exertion caused by improper lifting acrobatics. The bending angles of the elbow in volleyball are also measured by the flexible output piezoelectric sensor (Figure 3a) [38]. The athlete’s straight arm helps to rise the hitting point and increase the hitting power, making it easier to break through the opponent’s block defense. By capturing the voltage of the sensor (Figure 3b), a positive correlation with the bending angle of the elbow can be derived and used to evaluate the posture of different players. A similar strategy can also be found in measuring the bending angles of the wrist and knee during different spots. 

The monitoring of acceleration or magnitude of sporting motions is often realized by wearing inertial measurement units (IMUs) on the wrist/hand and shank/foot. As a faster, more reliable and cost-efficient strategy for activity and motion analysis, wearable IMUs benefit a lot from the significant reduction in sensor volume and price and are playing a more and more important role in the field of sports analytics. Commonly, an IMU consists of an accelerometer for measuring linear acceleration, a gyroscope for angular acceleration and sometimes a magnetometer for a magnetic field. Moreover, three-dimensional sensors are favorable due to their ability to capture parameters along the three axes and provide detailed and useful component data for orientation and kinematics studies. The combination of multifarious sensors ensures the system robustness and accuracy of captured data and then improves the validity and reliability of activity detection and analysis. The analysis of motion sequences in several sports (e.g., tennis, swimming, football, running, etc.) has been conducted in the past few years with the help of commercialized devices from different companies, such as Zepp, Actofit, Kinexon, Garmin and Motus [27]. In the meantime, researchers also use the tailored system to get more abundant information on sport motions [52,53,54]. For example, stroke detection and recognition of tennis can be realized by IMU sensors worn on the dominant arm or wrist [55,56]. In [57], two IMUs worn on the right arm and right leg of the player are used to detect the main events of breaststroke swimming. More recently, a pair of IMUs on the athletes’ lower back and hands are used to capture the acceleration and rotational speed signals in the motions of karate (Figure 3c) [58]. Relation analyses are conducted between the reverse punch temporal structure and the maximal hand velocity achieved by competitors. The performance evaluation in swimming phases, namely, wall push-off, glide, stroke preparation and swimming, of elite swimmers in different techniques (e.g., front crawl, breaststroke, butterfly and backstroke), is also realized by wearing an IMU on the waist (Figure 3d) [59]. However, most of the used sensors are developed using Micro-Electro-Mechanical System (MEMS) techniques. The miniaturization of MEMS sensors ensures the wearability, but the inherent rigidity and fragility of sensor chips inevitably damage the flexibility. Great efforts are still needed to develop these flexible inertial sensors. 

Shock impacts acting on the player are also an important issue in sports monitoring. A large impact beyond the tolerance threshold may cause damage to muscles, bones, soft tissues and inner organs. At present, impact detection in the sports domain mainly depends on the accelerometer, which interprets peak acceleration as a proxy for impact. Generally, the following two kinds of shocks often happen in sports: shock from own movements and impacts generated in confrontations [30,60]. Foot strikes in running and landing are the main form in single sports and have been monitored by the IMUs on the ankle or foot. Few studies also introduce plantar pressure sensors into the shock impact. In invasion and team sports such as football, lacrosse, rugby and soccer, the impacts on the head, body and limbs are much more acute, which is also monitored by the wearable IMUs. Due to the short-term and large amplitude of shock impacts, MEMS sensors play the dominant role in this field and the application of flexible accelerometers has become much rarer.

### 2.2. Physiological Indicators

Physiological indicators are very important parameters when evaluating the somatic function of athletes. According to the type of signal, the indicators can be divided into the following two categories: vital signs and metabolism parameters [24]. The former mainly includes heartbeat, breath, blood pressure, electrophysiological signals and body temperature, indicating the responses to exercise load, fatigue state and recovery level. The latter represents the changes in various biochemical indicators in the body. By monitoring the electrolytes and metabolites in body fluids, the physiological information of athletes, such as functional changes and energy metabolism can be well portrayed [61,62]. Nowadays, the real-time monitoring of vital signs and metabolism parameters has been greatly revolutionized by booming wearable electronics. 

#### 2.2.1. Vital Signs

- Heartbeat and breath

Heartbeat and breath are the two most important activities of human life. Breath is the only way for the body to get oxygen, and the heartbeat drives the nutrients and oxygen throughout the body. For breath monitoring, many efforts have been devoted to developing devices for sensing the airflow near the nostrils or mouth and the cavity volume variation in the chest/abdomen. Blood oxygen saturation, namely, SpO_2_, is a more effective and convenient to assess the body load and breathing efficiency of athletes during training or competition. SpO_2_ shows the proportion of oxyhemoglobin in blood hemoglobin and can be measured by the different light absorption capacities of oxyhemoglobin and deoxyhemoglobin. Many commercialized smartwatches have implanted the function of monitoring blood oxygen. To further improve the accuracy and comfort level, several flexible patches with organic light-emitting diodes (OLED) and photodiodes are also implemented by researchers. For a heartbeat, heart rate and its variability (HR and HRV) are two key values that provide clinical information about the health status of athletes [63,64,65]. The pulse flow of arterial blood induces a variation in the absorption of green-yellow light (wavelength about 500 nm) and then generates an AC signal synchronized with the systolic and diastolic activity of the heart. This phenomenon has been widely utilized to measure HR and HRV with the help of photoplethysmography (PPG) [66]. Comparatively, HRV is more helpful in improving an athlete’s cardiopulmonary function, especially in endurance training. A large HRV often indicates that the potential has not been fully exploited, while a small value may be associated with excessive fatigue. Properly adjusting the training schedule according to HR/HRV can effectively enhance the endurance of athletes.

- Electrophysiological signal

An electrophysiological signal refers to the sum of the electrical potentials generated by the cells and tissues in the human body. Common signals include electromyography (EMG), electrocardiogram (ECG) and electroencephalogram (EEG) [67,68,69,70]. EMG is produced by the contraction of muscles during exercise and can be used to evaluate the strength exerted by muscle tissues. The obtained information will be a very important basis for the optimization of training patterns and diagnosis or rehabilitation after injury. ECG contains information about heart functioning, from which unusual states can be detected in time, avoiding sudden cardiogenic attacks for athletes. EEG is from the electrophysiological activities of nerve cells in the brain and can be used to analyze emotion and sleep state. Currently, EMG is the most commonly used indicator in sports monitoring, especially in events with great explosiveness such as sprinting, jumping and gymnastics. The acquisition of electrophysiological signals is usually based on the needle or surface electrodes in specific regions, and the electrical signals are then transmitted to a collecting and analyzing system through wires or wireless communication. However, signal reliability is often affected by interference from intense exercises, which brings great difficulties in developing wearable monitoring systems with high precision and real-time capability.

- Body temperature

Body temperature is a key sign for human healthcare, and a too high or too low body temperature can be life-threatening. The changes in ambient temperature can lower the body’s thermoregulating function, which may cause a large deviation in the body’s core temperature and then weaken the athlete’s output power and endurance. These unfavorable factors can lead to a drop in sports performance, especially in events requiring endurance and team cooperation. By introducing the measuring units, the real-time monitoring of body temperature and its variation can provide an important reference for evaluating the thermal comfort level and effectiveness of acclimatization training before an event. Conventional thermometry methods cannot realize the continuous monitoring and temperature mapping ability, and some newly developed epidermal sensing arrays can be a proper solution to high-performance thermometry in sports [71,72,73,74].

#### 2.2.2. Metabolism

- Sweating loss

Monitoring the sweat rate (SR) or sweat loss (SL) of an athlete can provide information on sweat fluid to help optimize fluid replacement and minimize the problems related to body fluid imbalances during and after training/competition [75,76]. The planned amount of fluid for replacement should be customized based on the state of sweat loss. Meanwhile, fully monitoring SR is also critical for re-establishing euhydration, which needs a proper estimation of electrolyte losses. SR can be simply measured by the body mass changes over time, but this imprecise, belated method may not detect the appearance of fluid imbalances in time, which greatly affects the performance and even the health of players [77,78]. Moreover, the volume of sweat fluid varies considerably within athletes and can be affected by many factors, such as the following: climate, ventilation status, sporting equipment, the intensity and duration of exercises, etc. Therefore, many standardized sweat patch techniques are employed. A conventional patch consists of an absorbent pad and an adhesive dressing for occlusion. Before patching, the skin in the sampling region should be cleaned with deionized water and dried with gauze or paper. During exercise, the patch is infiltrated by sweat and removed before complete saturation. Then, the volume of sweat fluid was tested in a laboratory with the help of a centrifugal machine or syringe. This strategy improves the measuring accuracy, but the real-time capability is still limited. Recently, several flexible patches have been developed with microfluidics for sweat collection and colorimetric elements for sweat sensing. Some patches also deploy wireless transmission for long-distance, real-time monitoring [12,13,78,79,80,81].

- Metabolites and electrolytes

As an essential for life, the balance of biochemical metabolites and ions in body fluids plays a great role in keeping the body functioning properly. Variation in the concentrations of each physiological indicator can reflect the specific state of metabolism, energy supply and endurance. Generally, the common metabolites in sport monitoring include lactic acid and glucose. Lactic acid is the final product of anaerobic glycolysis, which originates from the insufficient oxygen supply during exercise [77,82,83,84,85]. The accumulation of lactic acid in the body can lead to muscle fatigue, pains and even lactic acidosis. Thus, the lactic acid level is an important indicator to evaluate the sport ability under anaerobic conditions and the intensity of training or competition. Glucose is an important energy supply for athletes under high-intensity exercises, and the glycogen-storing level in the body is a determining factor for athletes to perform high-intensity or prolonged exercises. By monitoring the changes in glucose concentration, the physical state of athletes can be evaluated in real-time and a rational training load-performance strategy can be built. Meanwhile, the balance of ions in the fluid also has detrimental effects on the functions of body tissues and organs. The excess loss of ions will affect the normal function of the human body and then decrease the sport performances. For instance, the lack of potassium ions may influence the heartbeat, and the excess loss of sodium ions can cause fatigue and cramps in muscles. Conventionally, the measurement of free ions and metabolites is conducted through blood sampling, which is invasive, time-consuming and suffering. The obtained results can only express the states before and after exercise, not the real-time and continuous data. With the progress of wearable devices, many non-invasive continuous monitoring approaches have been implemented to detect a variety of metabolites and electrolytes. Sweat, saliva and tears have been collected for evaluating the concentrations of glucose, lactic acid, uric acid and ions (e.g., Ca^2+^, Na^+^, K^+^, Cl^−^). Well-designed selectivity capacitates the multi-functionalization in some wearable flexible electrochemical sensors, making these platforms more powerful when simultaneously testing different electrolytes and metabolites.

## 3. Devices for Kinematics

As mentioned above, the detected kinematical indicators include body deformations and the velocity, acceleration and force accompanying these deformations. Correspondingly, the available devices mainly target measuring the strains induced by different deformations, associated pressure and inertial parameters. Subsequently, the sensors for monitoring these kinematical indicators are introduced in detail.

### 3.1. Strain Sensors

**Figure 4:**
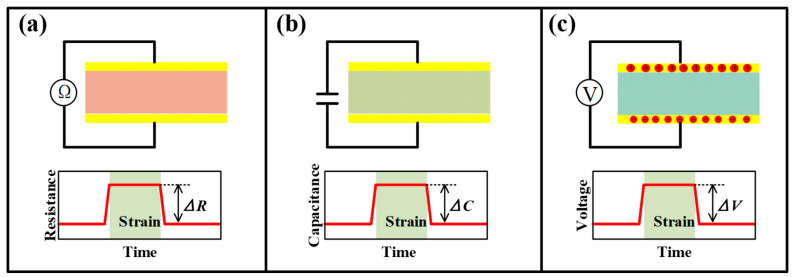
The illustration of mechanisms of (**a**) piezoresistive, (**b**) capacitive and (**c**) piezoelectric strain sensors.

Most movements in sports are realized by the deformation of joints, muscles and bones. These physical activities are bound to generate local strains, which can be detected by strain sensors to characterize the performances of players. Figure 4 shows common sensing mechanisms for strain sensors. Generally, the strain sensor mainly relies on the piezoresistive mechanism to transform the captured strain into the resistance change of the functional element. In addition, capacitive and piezoelectric mechanisms are also used by several devices to output capacitance and voltage signals when triggered by strains.

The resistance change of conventional elements in piezoresistive strain sensors is mainly due to changes in the shape or material resistivity [86]. The former is the dominant mechanism for metal strain gauges, and the latter is commonly used in silicon-based MEMS piezoresistive sensors. For flexible piezoresistive sensors, it is not easy to define which factor dominates the resistance variation due to the joint participation of microparticles and macroscopic deformable materials in many devices. However, it is a shared goal in developing different kinds of sensors that pursue a great sensitivity coexisting in the wide-range device. Especially for sports with large strains, measuring range is a critical factor that must be considered. With the emerging progress of structural engineering and fabrication techniques, many ingenious schemes are utilized to improve both measurement sensitivity and range for flexible strain sensors. 

**Figure 5:**
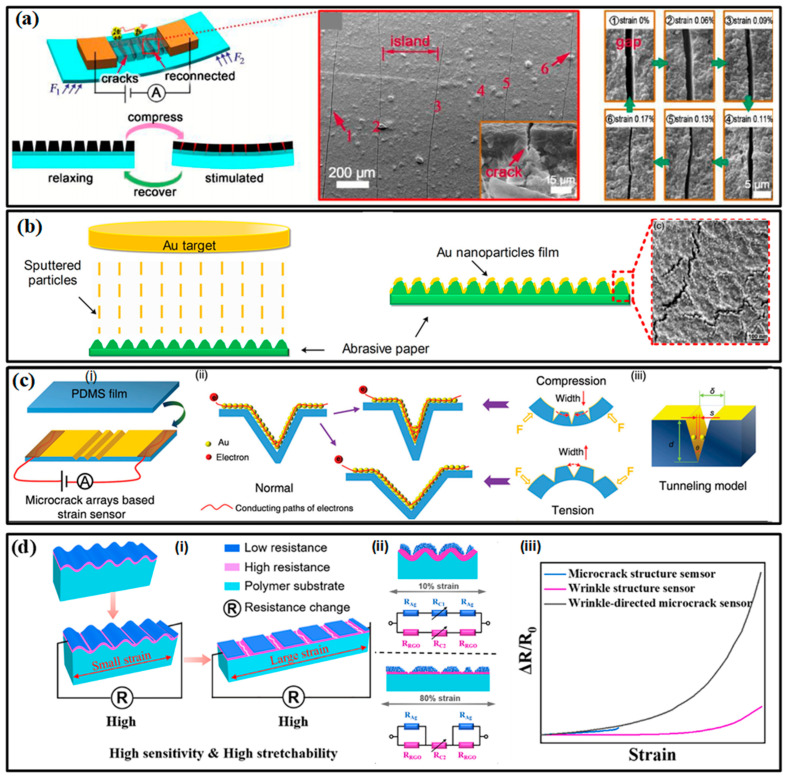
The high-performance strain sensors: (**a**) the crack-based strain sensor [88]; (**b**) the strain sensor made of abrasive paper and cracked Au film [89]; (**c**) the strain sensor with tailored V-notches on PDMS [90]; (**d**) the winkle-cracked strain sensor. (i) the operating mechanisms under small and large strains; (ii) is the circuit model of the sensor under 10% and 80% strain; (iii) the comparison between three kinds of strain sensors. [91]. Reproduced with permissions from The Royal Society of Chemistry (2017) [88]. Reproduced with permissions from American Chemical Society (2017) [89]. Reproduced with permissions from The Royal Society of Chemistry (2018) [90]. Reproduced with permissions from Elsevier (2022) [91].

Micro-scale margins for deformation are reserved to meet the requirements of large-range tests. Serpentines, micro-wrinkles, overlapped structures and micro-porousness have proved their significant efficacy in increasing the sensing range. These special configurations can increase the stretchability and reduce the deformation resistance of the structure, allowing the sensor to withstand a larger strain range without being damaged. Most engineered strain sensors can capture strains at around 100%. A porously structured device by Zhou et al. has realized a test range up to 950% with a favorable gauge factor (GF) of 364.5 [87]. Although the tunneling effect of many polymer-filler composites already provides favorable sensitivity, the participation of sophisticated structures is also required to further improve the GF. Taking the crack-based sensor in Figure 5a as an example, the opening and closing motions of crack scarps on thin conductive films will change the cross-sectional area of the conductive path and then affect the resistance of the whole film, allowing the sensors to detect subtle strain stimulus [88]. Moreover, when the separated scarps are on the nanometer scale, the tunneling effect may also exist, giving the electrons a probability to flow across the gap. A nonlinear input-output relationship can be found in most of the crack-based strain sensors. When the strain continues to grow (often larger than 1%), all cracks may be fully opened and the sensing performance also degrades until it disappears. A structurally engineered substrate with strain concentration structures can control the location of the crack and accelerate its propagation, which further improves the sensitivity. For example, abrasive paper pieces have been utilized as a substrate for depositing Au film to form flexible bending strain sensors (Figure 5b) [89]. Some more sophisticated microscale concentration structures, e.g., the tailored V-notches on polydimethylsiloxane (PDMS) film in Figure 5c, can give the strain sensor a GF of 5888.59 at a strain of about 2% [90]. However, it is not easy work to realize a wide-ranged, highly sensitive strain sensor based on only one enhancing method due to the contradiction between the two parameters. Therefore, the hierarchical structures were developed for the improvement of both sensitivity and sensing range. More recently, Li and co-workers introduced both wrinkles and cracks into a strain-sensitive fiber by exploiting a new strain-sensing bilayer consisting of a pre-wrinkled rGO/PMDS layer and a highly susceptible AgNPs layer (Figure 5d) [91]. The robust rGO interlayer cracks the Ag film at wrinkling troughs, and the stress-relief wrinkling characteristic offers the bilayer excellent stretchability. The obtained strain sensor features a large test range (about 210%) and unprecedented GF both in subtle and large strain ranges (0–2%, GF: 420; 110–125%, GF: 1.1 × 10^9^).

**Figure 6:**
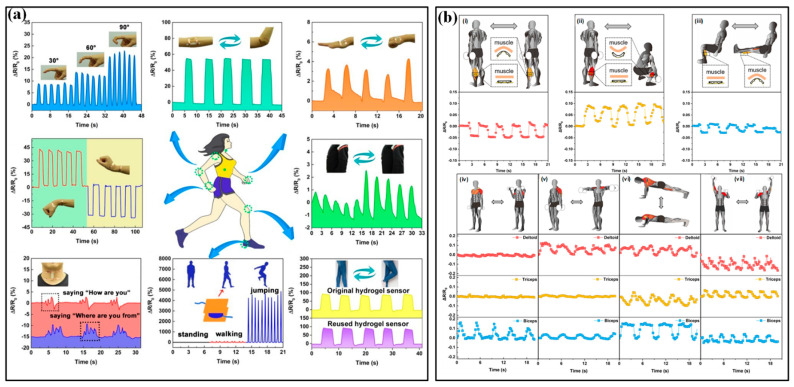
The applications of strain sensors in sport monitoring: (**a**) monitoring the joint bending of different parts of body [92]; (**b**) monitoring the muscle contractions during extensions of standing heel lifts (i), squats (ii) and leg (iii) and the state of biceps, triceps and deltoid during standing dumbbell curls (iv), side lateral raise (v), push-ups (vi), and shoulder press (vii) [93]. Reproduced with permissions from The Royal Society of Chemistry (2020) [92].

Inspired by the excellent sensing features, strain sensors have played a very important role in monitoring body motions. Generally, human motion depends on the combined action of skeletal muscles, bones and joints. Joint bending usually induces large strains on the corresponding epidermis, which has been successfully tested by a group of piezoresistive strain sensors (Figure 6a) [92]. The distinguished activities include dynamic bending movements in the forefinger, elbow, wrist and knee; making a fist in hand and postures of standing, walking and jumping. In addition to these palpable bendings, many subtle variations induced by muscles also receive excellent monitoring. For instance, the vibrations of the vocal cords in the larynx are accurately perceived when speaking the words “How are you” and “Where are you from”. Muscle contractions are an inevitable part of the exertion in sports and only lead to subtle stretching or contracting strains in the corresponding epidermis. Benefitting from the high sensitivity and high resolution of many elaborate strain sensors, these varied, subtle muscle activities can be easily detected by mounting these strips on skins. As shown in Figure 6b, a kind of fiber-junction bending sensor by Li et al. has completed this task with high quality [93]. The sensor on the epidermis corresponding to calf muscles can identify the magnitude and direction of epidermis strains in three sets of fitness actions, including standing heel lifts, squats and leg extensions (Figure 6b(i–iii)). Based on the captured electrical signal, the squat led to a more significant deformation than the other two fitness actions. Squatting generates a contract strain in the calf muscles and a stretch strain appears when heel lifting and leg extensions are performed, which is the same as the phenomenon observed in real-time videos. Moreover, multichannel monitoring of contractions in different muscles is also investigated (Figure 6b(iv–vii)). Three sensors were attached near the bicep, tricep and deltoid muscles and then simultaneously detected the muscle contractions caused by standing dumbbell curls, side lateral raises, push-ups and shoulder presses. During strain-based monitoring, the crosstalk from external pressure/force may influence the accuracy of obtained results. It is possible to locate the devices in the region that is free from interference, but the limited candidates cannot fulfill the whole monitoring work. A more potential method is making the sensor insensitive to the external undesirable pressure/force. Recently, Xu et al. reported a novel flexible tensile strain sensor that could decouple the simultaneously loaded pressure and was insensitive to the external load-induced off-axis deformations [94]. This integrated sensor patch has successfully detected the activities of fingers and wrists. 

### 3.2. Pressure Sensors

Pressure can reflect the contact state between an athlete and the outside, and the available measuring device is a flexible pressure sensor for testing the contact area and contact force. The sensing principle of a flexible pressure sensor is similar to that of the aforementioned strain sensor, mainly including piezoresistive, capacitive and piezoelectric. In addition, some new mechanisms, such as triboelectric, are also being applied. Generally, a pressure sensor consists of an electrode layer to transmit signals and an active layer that deforms under pressure and dictates changes in output signals. In order to promote the performance parameters, such as sensitivity, range and limit of detection, response and relaxation speed, microengineered structures are often introduced. 

**Figure 7:**
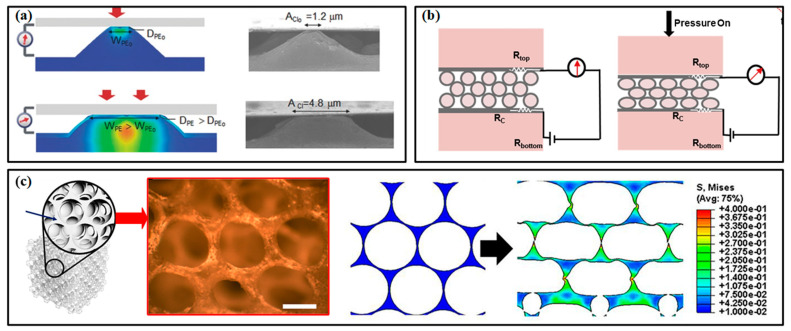
The high-performance pressure sensors with engineered structures: (**a**) pressure sensor with micropatterned layer [96]; (**b**) pressure sensor with multilayered structure; (**c**) pressure sensor with porous layer [98]. Reproduced with permissions from Wiley (2014) [96]. Reproduced with permissions from Elsevier (2018) [97]. Reproduced with permissions from American Chemical Society (2019) [98].

In piezoresistive pressure sensors, micropatterned structures, porous layers and multilayered structures have proved their superiority in improving the sensitivity and responding speed [95]. As shown in Figure 7a [96], the deformation of the micropatterned active layer under pressure can more distinctly change the contact area between it and the electrode, which inevitably varies the whole resistance of the device and then improves the sensitivity. Pyramids, domes and semi-cylinders have been widely used for micropatterning the active layer. The semi-cylinder lines can generate a significantly higher rate of increase in contact area than the other two microstructures within the pressure range of 0–2 kPa. When compressed by higher pressure, the deformation of the microstructure tends to saturate, resulting in a decrease in sensitivity. The multilayered structure can be realized by layering conductive microspheres, in which the variation of contact area can be attributed to the deformation between the microspheres and electrodes (Figure 7b) [97]. The porous layer strategy can change the modulus of the active layer (Figure 7c) [98]. Due to the air voids among these microstructures, the active layer tends to deform and recover more easily. A larger pore can improve the sensitivity, but the more deformable active layer can induce a decrease in the dynamic range of sensors. This phenomenon improves the response and recovery time of sensors and lowers the detection limit. However, it is worth noting that there is no universal optimal design for all piezoresistive pressure sensors, and it may be a tailored process to develop a sensor for certain applications. 

In capacitive pressure sensors, manipulating the geometry of pressure-sensitive materials is also a practical method to enhance performance [99]. Generally, the compressibility of the dielectric layer is increased by adding voids through microengineering. The interelectrode distance and the dielectric constant of the media can then be changed more easily under a given applied pressure. The micropatterned and porous structures in dielectric layer are two often used approaches [100,101]. Micropatterns decrease the stiffness of the dielectric film due to the added air voids, facilitating the reduction of interelectrode distance and then improving the sensing performance. Moreover, the compressing process can extrude air (whose dielectric constant is lower than elastomer) from the device and further increase the sensitivity. Similar circumstances also happen in the dielectric layer with porous structures [102]. The increased compressibility and varied dielectric constant induced by the 1–1000 µm air voids bring a larger capacitance variation (consequently sensitivity), faster response and a smaller detection limit [99]. The approaches combining micropatterns and porosity in the dielectric layer are also proposed to further improve sensitivity. An accompanied consequence of micropatterns and porous structures is the decrease in measurement range, which may be overcome by using a thicker dielectric layer or tailored materials/structures that stiffen at higher pressures. 

Piezoelectric and triboelectric pressure sensors also benefit from the enhancement of microengineering. For instance, the micropatterned PVDF film can improve the output power by about 500% compared to that of the device with a flat film when a 15 kPa pressure is applied [103]. Meanwhile, the roles of micropatterns in triboelectric pressure sensors have not been largely explored, but several recent works have demonstrated their potential.

**Figure 8:**
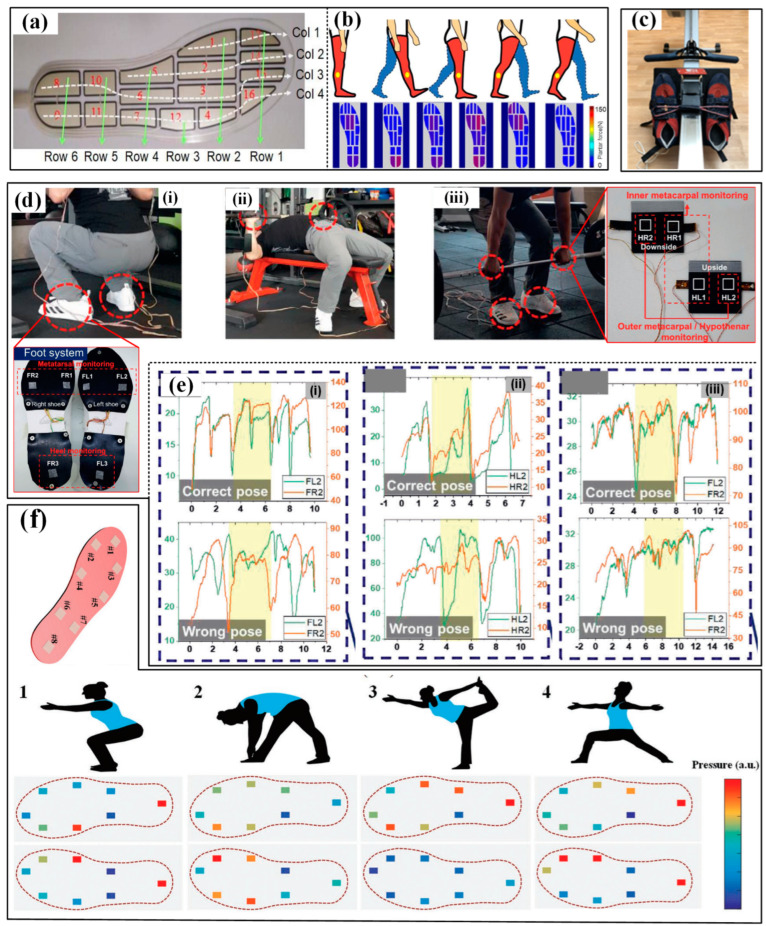
The applications of pressure sensors in sport monitoring: (**a**) detect and analyze the plantar pressure during walking and (**b**) the captured results [104]; (**c**) measuring the plantar pressure on ergometer [105]; (**d**) monitoring the pressures on hand and foot during powerlifting in three different ways (i–iii) and (**e**) the obtained pressure results for right or wrong poses for the corresponding ways [31]; (**f**) monitoring the pressure distribution under four yoga postures [106]. Reproduced with permissions from Wiley (2021) [31]. Reproduced with permissions from Wiley (2019) [106].

The most popular application of pressure sensors is monitoring the plantar pressure during sports by directly integrating it into a shoe insole. Firstly, monitoring the plantar pressure is a very important path to evaluating the gait of athletes. Several gait variables during sports have been obtained, including peak/mean pressure, reaction force, the center of pressure (COP), the distance between COP and the contact region/area. Zhao et al. used an insole-shaped flexible sensor matrix film with 16 piezoresistive sensing cells to detect and analyze the plantar pressure (Figure 8a) [104]. The measured total static pressure forces (455 N) of the right (232 N) and left feet (223 N) were similar to those while standing on one foot for the right foot (450.7 N) or left foot (456.7 N) when the subject was in a natural upright standing position. The variation profiles of the plantar pressure force during a dynamic gait cycle were also measured, including the stages of heel strike, foot flat, midstance, heel off and toe off midswing (Figure 8b). Barratt et al. measured the plantar pressure and reaction force of two commercial sensing insoles while participants were rowing on a Concept2 ergometer (Figure 8c) [105]. The results showed that the Moticon and Pedar-x insoles had moderate-excellent test–retest reliability, but the former was not suitable for accurately measuring pressure and force variables over time due to its overestimation. Recently, Jeong et al. proposed an ultra-wide range pressure sensor based on a piezoresistive microstructured nanocomposite. The wide pressure range, favorable sensitivity and high durability made the developed sensor suitable for monitoring the pressure distribution in the hands and feet during powerlifting workouts (Figure 8d) [31]. Wrong poses, including the imbalance between left and right hands, unstable dynamics induced by perturbation between right and left feet, and imbalance in hand and foot motions caused by pelvic deflection, were successfully discovered and confirmed by a certified personal trainer (Figure 8e). Similarly, a textile-based wireless pressure sensor was integrated into an insole to monitor the pressure distribution when the participants were doing yoga postures (Figure 8f) [106]. In the meantime, the data measured by pressure sensors can also represent the contact between facilities and hands, and the involved devices are often referred to as artificial electronic skin that mimics the tactile pressure sensitivity of human skin. One typical prototype is the e-skin-based prosthetic hand demonstrated by Kim et al. The integrated piezoresistive pressure sensors can reliably transduce signals from typing and grasping the baseball and then transfer them to the nervous system by connecting the sensors with the nerve. Yang et al. also used a flexible triboelectric nanogenerator in skiing [50]. The nanogenerator can be easily manufactured in different shapes or structures and applied to the insole or ski pole sleeve to monitor the pressure distribution.

### 3.3. Inertial Sensors

Generally, inertial sensors for kinematics monitoring in sports often work as an inertial measurement unit (IMU), which measures linear acceleration, angular acceleration and sometimes magnetic field. The accelerometer measures the time derivative of velocity (namely, linear acceleration) so that they can be used for kinematics study and orientation using acceleration components in 3D space; the gyroscope measures angular acceleration about a certain axis and can be used to determine orientation in the angular coordinate system; the magnetometer measures magnetic field strength. The combination of the three sensors can produce a measuring system for activity detection and analysis. As for now, inherently flexible accelerometers and gyroscopes are not as common as the abovementioned devices. Only a few prototypes are reported, whose performances are quite far away from those of commercialized MEMS devices. Thus, most wearable IMUs use flexible substrates to integrate the miniaturized solid sensors into flexible systems with wearability. Herein, the involved inertial sensors may be individual units with only one function and monolithic integrated systems with multiple measuring capacities. Moreover, an MCU is fused into some high-performance systems to enhance signal transmission and processing capability. These approaches provide a strong base for monitoring the inertial kinematics parameters in sports. A mass of commercialized wearable IMUs have appeared on the market and more information can be found in [27]. 

**Figure 9:**
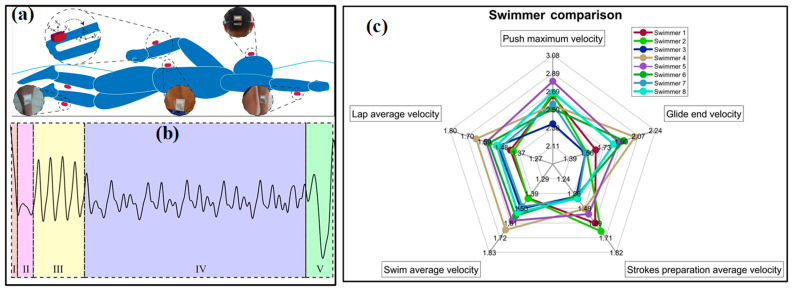
The applications of IMUs in sport monitoring:(**a**) analyze the swimming phases of swimmers and (**b**) obtained signals from wall to wall (I-push-off, glide, II-strokes preparation, III-IV-swimming and V-turn) [36]; (**c**) a result from the coaching assistance system “Smartswim” [110].

As for the applications of wearable IMUs in sports, the target information focuses on posture/motion and impact [107,108,109]. The former can be a very important assistant for a coach to classify the movements and conduct skill evaluation and acquisition for players. For example, in some racquet sports, e.g., tennis and badminton, IMUs have been mounted on the forearm, wrist or bottom of racquets to acquire data for recognizing the strokes and classifying them into serve, backhand and forehand. As shown in Figure 9a,b, the authors of [36] use six IMUs worn on shanks, wrists, sacrum and head to analyze the swimming phases of swimmers from wall to wall (namely, wall push-off, glide, stroke preparation, swimming and turn). The results proved that the sacrum is the most appropriate location for implementing a single sensor analysis system, which is verified by a further performance evaluation using a single IMU in the main swimming postures [59]. With the obtained data, a coaching assistance system, “Smartswim” is proposed to quantitatively assess swimmers’ performance and lead to more efficient training (Figure 9c) [110]. The identification of running asymmetry is another practical domain for wearable IMUs. The participation of IMU in monitoring running posture and identifying asymmetry has been evaluated and verified. Moran et al. used a wearable IMU sensing system to identify the running asymmetry of 21 participants with an artificially induced asymmetry [40]. Moreover, multiple IMUs are mounted on different parts of the body to get the optimal location. Many works have proved that the IMUs on lower limbs, such as the foot, heel, knee and hip, can realize better performance in capturing kinematical parameters. In invasion sports (e.g., soccer, football, rugby and basketball), the skilled motions and high-level impacts should be equally monitored [111]. The motions can be recorded by the IMUs mounted on the body [112,113,114,115,116], and the adversarial impact is usually captured by accelerometers. The accelerometers can be directly worn by players and to capture trunk collisions, and some instrumented equipment, such as helmets, headgear and mouthguards, is also utilized to investigate the head impacts in these contact, collision and combat sports [60].

## 4. Devices for Physiologies

### 4.1. Devices for Vital Signs

#### 4.1.1. Heart Beating/Pulse

**Figure 10:**
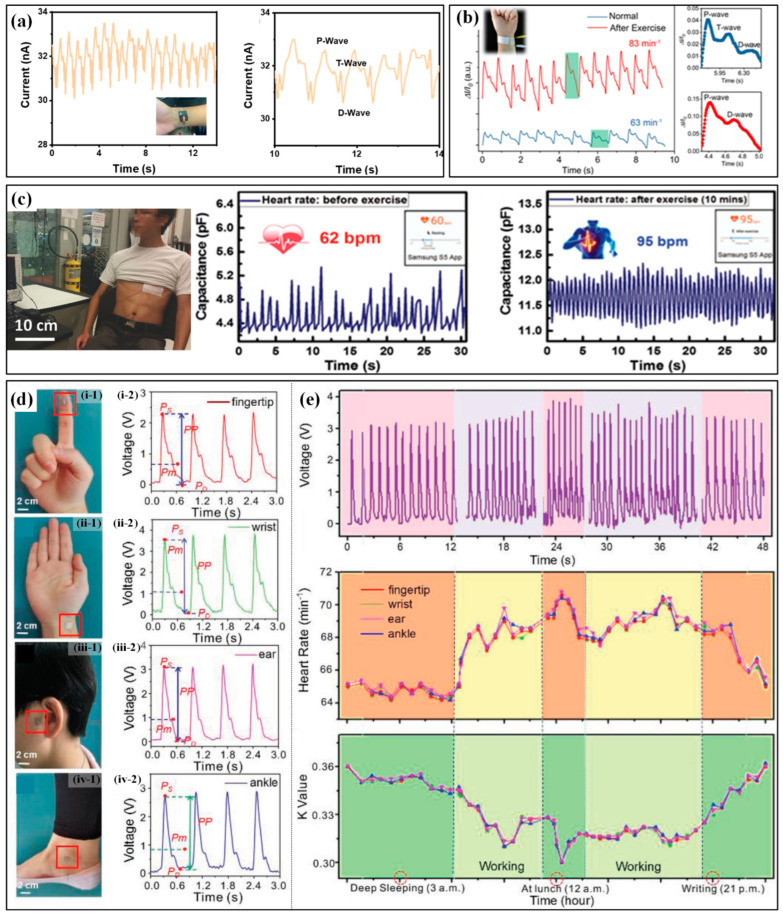
The wearable sensors and corresponding applications in monitoring heartbeat or pulse: (**a**) the pulse curve captured from wrist and P/T/D waves [117]; (**b**) the captured pulse signal and P/T/D waves before and after exercise [119]; (**c**) the captured heartbeat curve and heart rate captured from chest before and after a10-min running [120]; (**d**) the self-powered pressure sensors worn on different parts of body (i–iv) for monitoring heartbeat signals and the (**e**) captured voltage (up), heart rate (middle) and K value (down) in different segments of a day [121]. Reproduced with permissions from American Chemical Society (2022) [117]. Reproduced with permissions from American Chemical Society (2018) [119]. Reproduced with permissions from Wiley (2017) [120]. Reproduced with permissions from Wiley (2018) [121].

The monitoring of heartbeat and pulse can be implemented by measuring the accompanying physical parameters caused by these activities, which can be detected by highly sensitive strain or pressure sensors. Pulse detection methods can be categorized according to the location of the mounted sensor. The commonly used ones include CA (carotid artery), DA (digital artery), RA (radial artery), BA (brachial artery) and DPA (dorsalis pedis artery). Based on the structure of pine trees and needles in nature, Yu et al. proposed a self-powered piezoresistive sensor with zinc oxide nanorod arrays on graphene-treated cotton [117]. When worn on the wrist (RA), the sensor can successfully indicate the pulse by the occurrence of generated current peaks with an acceptable deviation in heart rate between the sensor and a commercial smartwatch. When further refining the heart rate-current response, the P/T/D waves are also detected (Figure 10a). The variation of pulse rate before and during exercises are detected by the highly sensitive graphene strain sensor on the wrist [118]. Chen et al. proposed a pressure sensor based on a piece of carbonized crepe paper [119]. As can be seen in the obtained waveforms (Figure 10b), the pulse rates rise in a normal state and after exercise are approximately 63 and 83 bpm, respectively. Moreover, the pressure sensor can also distinguish characteristic peaks of the pulse waveform, which are assigned to the P (percussion) wave, T (tidal) wave and D (diastolic) wave. Nassar et al. also successfully illustrated the detection of the heart rate rising from 62 bpm to 95 bpm after a 10-min run by a paper-based capacitive sensor when worn on the chest near heart (Figure 10c) [120]. As long as the performance meets the monitoring requirements, the sensor can be mounted on different positions of the body to detect the pulse. The self-powered pressure sensor proposed by Prof. Zhong Lin Wang’s group has been directly worn at the fingertip, wrist, ear and ankle, respectively, to continuously monitor the heartbeat signals (Figure 10d) [121]. Several consecutive period pulse waveforms were captured in different segments of a day, including deep sleeping, working, at lunch and night writing (Figure 10e). Based on the waveforms, the heart rate and K value are successfully calculated, and the measured heart rates from different positions show a favorable consistency. Moreover, the K value reflects the change in the characteristics and the area of the pulse transit map, which renders the degree of vascular sclerosis and then the cardiovascular physiology and pathology. Further on, a user-friendly system has been developed with the capability of signal processing, sampling and Bluetooth-based wireless communication. The real-time data can be received and displayed on a mobile phone APP. This is of great significance for the low-cost, real-time assessment of athletes’ heart status and the prevention of cardiac problems such as sudden arrests in sports.

**Figure 11:**
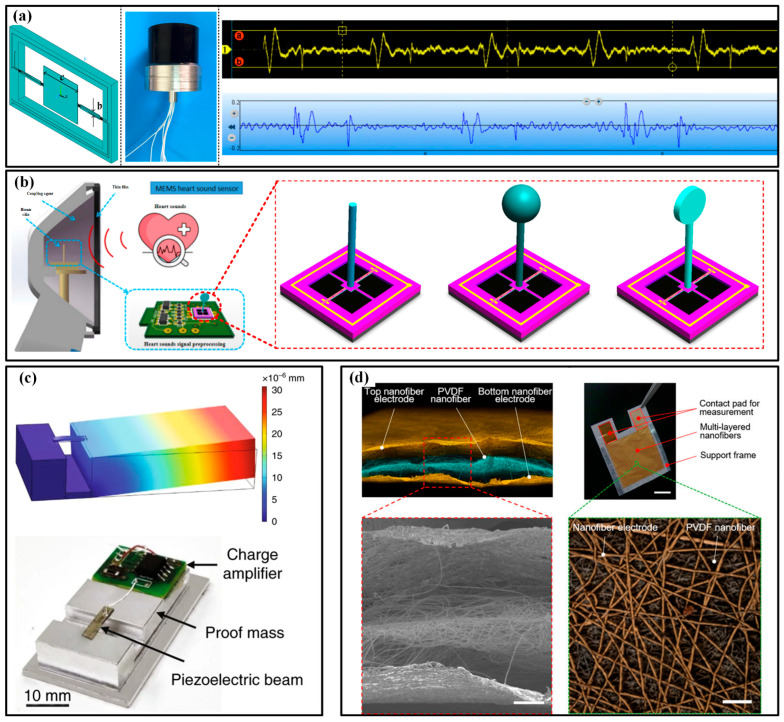
The PPG devices and their applications in monitoring heartbeat: (**a**) the double-beam-block microstructure(left), sensor photo (middle) and comparison of obtained curves (right) between this sensor (yellow) and commercial product (blue) [122]; (**b**) fish cilium structures for PPG sensors [126]; (**c**) the PZT-based sensor with a two-stage amplifier [129]; (**d**) the sensor based PVDF nanofibers [130]. Reproduced with permissions from Elsevier (2022) [126]. Reproduced with permissions from The National Academy of Sciences (2020) [130].

The heartbeat can also be recorded by phonocardiogram (PCG) and ECG. PCG devices record the mechanical heart sound signal to render the valve function and hemodynamics in the heart. By now, several electronic stethoscopes have been developed. The available devices guarantee the miniaturization of the system, and flexibility can be achieved with the help of flexible substrates. As shown in Figure 11a, the group of Wendong Zhang proposed a double-beam-block microstructure to combine the piezoresistive sensor with the natural frequency response of the heart sound (20~600 Hz) [122]. Compared with a commercial electronic stethoscope, the MEMS electronic heart sound sensor shows competitive sensitivity and a significantly higher signal-to-noise ratio (SNR). The MEMS heart sound sensor can provide the first and second heart sounds, containing more abundant information about the lesion. Meantime, the bat shape beam, T-type and crossbeam structures with a rigid plastic cylinder at the layout center that imitates the fish cilium are designed to detect the heart sounds with better sensitivity (Figure 11b) [123,124,125,126,127]. Except for the piezoresistive principle, some piezoelectric devices are also involved in this task. Ning et al. reported a triangular cantilever piezoelectric bimorph MEMS transducer to monitor the PCG [128]. The results show that the SNR of the MEMS stethoscope is 17 dB, approximately 10 dB higher than that of the commercial stethoscope, which can be further improved if an application-specific integrated circuit is integrated. Similarly, a PZT-based sensor with a two-stage amplifier is used to assess the heart states of pneumonia patients and successfully tracks the recovery course of the discharged pneumonia patients (Figure 11c) [129]. However, these devices are all made of a silicon wafer, inevitably resulting in inherent rigidity for these chips. To achieve the desired flexibility or wearability, some assisted components, e.g., bonds or fabrics, are needed to provide a substrate for integration. As for the devices with inherent flexibility, there are only a few works that conform to this characteristic. As a leading pioneer, Takao Someya’s team produced a piezoelectric mechanical acoustic sensor based on PVDF nanofibers by an electrospinning process (Figure 11d) [130]. The high SNR of 40.9 dB ensures the measuring accuracy and the lightweight (about 5 mg), excellent gas permeability (12.4 kg·m^−2^·d^−1^) and mechanically robust against repetitive bending (more than 1000 cycles) ensure comfort in long-term wearing.

**Figure 12:**
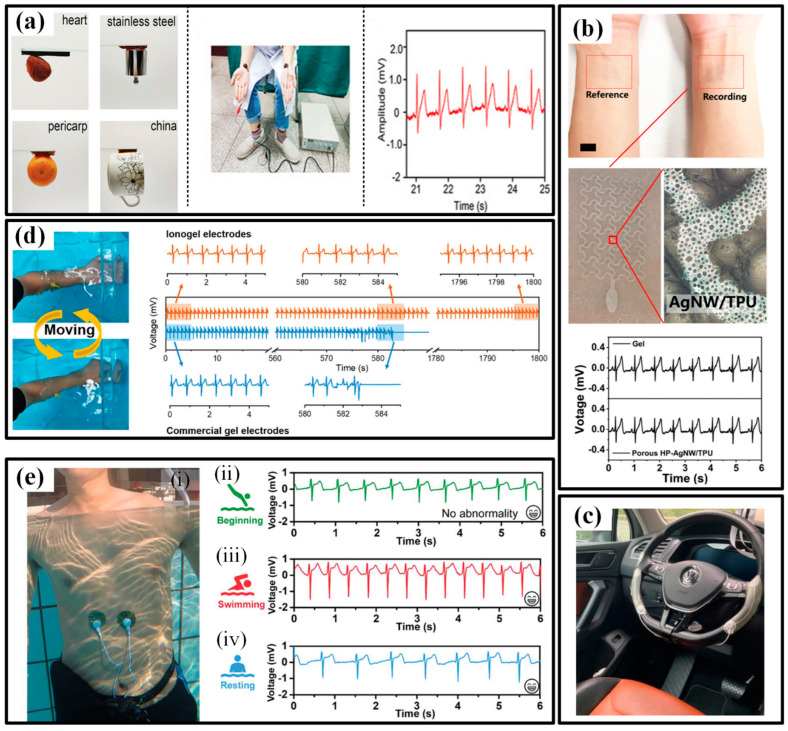
The wearable ECG devices and their applications in sport monitoring: (**a**) the electrodes with long-term and repeatable adhesiveness (left) and the electrodes worn on forearms (middle) to capture ECG signals (left) [70]; (**b**) the electrodes with porousness for excellent permeability (up) and the captured ECG signals (down) [136]; (**c**) the steering wheel with flexible ECG electrodes [137]; (**d**) the water-resistant electrodes and the long-term reliability underwater [141]; (**e**) the water-resistant conformal hybrid electrode for aquatic endurable ECG monitoring during swimming [32]. Reproduced with permissions from Wiley (2020) [32]. Reproduced with permissions from Wiley (2019) [70]. Reproduced with permissions from American Chemical Society (2020) [136]. Reproduced with permissions from Wiley (2021) [141].

An ECG is another typical signal for assessing the heart state. The research of available wearable devices mainly focuses on pursuing flexible, dry electrodes that can reliably acquire ECG without conductive glue [131]. Numerous high-conductivity nanomaterials, such as metals, carbons and polymers, are processed to guarantee reliable contact between electrodes and skin in various environments. In the beginning, the efforts were devoted to finding candidates to replace the conventional Ag/AgCl electrode. Pencil lead and liquid metal ink are utilized to provide drawability and simplified installation to ECG electrodes [132,133]. The results even prove that the pencil lead-based electrodes could acquire better ECG signals when used under freshwater or saltwater. Then, more and more functional materials are participating in the flexible electrodes to provide favorable features. Gan et al. prepared a super-stretchable, conductive and adhesive hydrogel by incorporating the PSGO-PEDOT nanosheets into a polyacrylamide hydrogel network. Due to the mussel-inspired redox environment inside the hydrogel networks, the obtained electrodes feature a long-term and repeatable adhesiveness, which significantly simplifies the ECG tests (Figure 12a) [70]. Bao’s group compounded a new flexible self-healing material with carbon nanotubes (CNTs) or Ag nanowires (NWs) to obtain a flexible conductive electrode with self-healing capability, possessing great potential in high-viable ECG electrodes [134]. To improve the permeability, porousness is introduced into the electrode geometries [130,135]. Zhou et al. employed a breath figure method to generate the porous skeleton before AgNWs are dip-coated, and the resulting film had a transmittance of 61%, the sheet resistance of 7.3 Ω/sq and water vapor permeability of 23 mg/(cm^2^·h) [136]. The ultrathin electrode forms a conformal contact with the skin and successfully captures the ECG signals (Figure 12b). Some other researchers are trying their best to extend the application range of flexible electrodes. As shown in Figure 12c, Warnecke et al. attached the printed flexible ECG electrodes to the steering wheel to continuously monitor heart health during driving in different situations, such as rest, city, highway and rural [137]. To maintain functionality underwater, many groups have introduced waterproof capacity into flexible electronics [32,138,139,140]. For instance, Wu’s group prepared an ionogel by a facile one-step polymerization and used it as water-resistant electrodes [141]. The stretchability, conductivity, underwater self-heal ability, underwater adhesiveness and biocompatibility make the ionogel possible to be used as bioelectrodes for underwater ECG monitoring. When the electrodes are continuously shaken underwater with a human forearm, the commercial gel electrode loses the ECG signal within 10 min, but the ionogel electrode is able to record ECG signals continuously for 30 min underwater (Figure 12d). Moreover, the ionogel electrode can maintain its conductivity and mechanical properties and effectively detect ECG signals even after 14 days of immersion in water. Similarly, Ji et al. reported a water-resistant conformal hybrid electrode for aquatic endurable ECG monitoring and achieved the real-time recording of ECG signals during swimming (Figure 12e) [32].

#### 4.1.2. Respiration

The monitoring of breath is mainly based on the following four approaches: (i) measuring the variation of humidity near the nostril or mouth; (ii) detecting the deformation of the thorax; (iii) monitoring the air pressure near the nostril or mouth; (iv) measuring the blood oxygen to assess the efficiency of respiration. 

**Figure 13:**
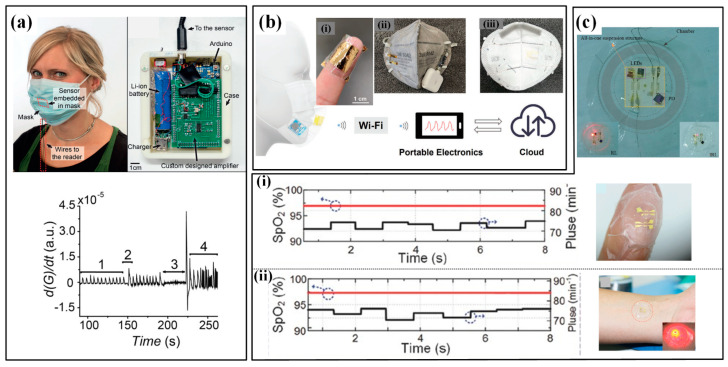
The wearable respiration sensors: (**a**) the face mask with a paper capacitive moisture sensor and corresponding circuit for recognizing breath patterns [144]; (**b**) smart face mask based on a self-powered ultrathin pressure sensor for wirelessly monitoring breath. (i) is the ultrathin sensor twining one a finger; (ii) and (iii) are the front and back sides of the smart face mask [33]; (**c**) epidermal inorganic optoelectronic SpO_2_ sensor and results measured from fingertip (i) and wrist (ii) [148]. Reproduced with permissions from Wiley (2022) [33]. Reproduced with permissions from Wiley (2016) [144]. Reproduced with permissions from Wiley (2017) [148].

The transient difference of moisture in inhaled and exhaled air can induce an obvious humidity variation, which can be detected by highly sensitive humidity sensors [142,143]. Whitesides’ group proposed a paper-based capacitive moisture sensor that uses the hygroscopic character of paper cellulose to measure the respiration patterns and rate by digitally printing graphite ink on a paper sheet (Figure 13a) [144]. The paper sensor can be embedded into a face mask and its outputs are processed and transmitted to recognize different breath patterns such as normal breathing (1), taking a deep breath (2), pausing (3) and randomly breathing (4). Similarly, Simić et al. proposed a textile capacitive facemask sensor with interdigitated electrodes to measure the humidity variation by testing the permittivity change due to the humidity [145]. The interdigitated electrodes are directly embroidered onto the inner or outer sides of the medical mask, which greatly improves the system integration and portability. 

The middle two routes mainly focus on the physical parameters induced by breath. Takao Someya’s team proposed a smart face mask based on a self-powered ultrathin pressure sensor for wirelessly monitoring breath (Figure 13b) [33]. The sensor consists of two Au/parylene/Teflon AF films and works based on the mechanism of the electrostatic induction effect. The sensor possesses the thinnest thickness of about 5.5 µm and the lightest weight of about 4.5 mg and can achieve a peak open-circuit voltage of up to about 10 V when stimulated by the airflow of breath. With the help of a measuring circuit, different breathing conditions, including normal breathing, fast breathing, coughing and breath-holding, are recognized. More recently, Karita et al. developed a wearable sensor for respiration monitoring during 6-min walk by sensing the variation in the capacitance of abdominal skin [146]. The conductive cloth electrodes are sewn inside the belly band, and two configurations of left-right layout and coaxial layout are utilized to capture the capacitive signals. 

Blood oxygen is often measured by optoelectronic devices based on the PPG method [147]. Li et al. prepared an epidermal inorganic optoelectronic device by integrating III–V group emitting elements, a Si-based photodetector and interconnects (Figure 13c) [148]. Because of the superior flexibility/stretchability, this device can be conformably mounted to skin and keeps the constant light transmission between emitting element and photodetector. When attached to the forefinger or wrist, the SpO_2_ and pulse rate are successfully measured. Moreover, Chen et al. proposed a flexible blood oxygen monitor with a power source of a triboelectric nanogenerator, which provided the potential for battery-free wearable electronics [149]. 

#### 4.1.3. EMG and EEG

**Figure 14:**
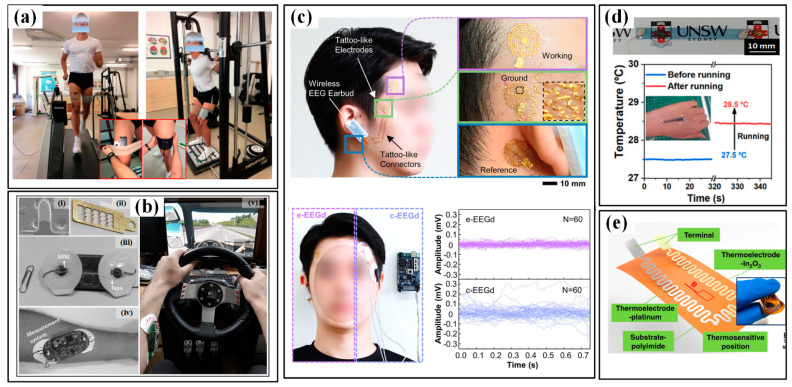
The EMG, EEG and temperature sensors for sport monitoring: (**a**) the EMG sensor to monitor the strength and endurance exercises in vivo [150]; (**b**) the microneedle array electrode-based wearable EMG system to detect the driver drowsiness. (i) is the SEM photo of one single needle; (ii) is the photo of the microneedle array electrode; (iii) is the wearable EMG system; (iv,v) are the system worn on forearm and driving [151]; (**c**) the earbud-like wireless EEG device (up) show a good ability in decreasing direct noise (down) [152]; (**d**) the wearable temperature sensor (up) and the measured small rise of skin temperature before and after a 5-min running exercise (down) [153]; (**e**) thin thermocouples film [39]. Reproduced with permissions from American Chemical Society (2020) [153].

The utilized electrodes in the detection of EMG and EEG are similar to ECG. Therefore, the special requirements of electrodes are also similar to those of ECG, such as flexibility, water resistance and durability, etc. For instance, Giminiani et al. showed a fabric-based thigh-wearable EMG sensor for monitoring quadriceps activity during strength and endurance exercises (Figure 14a) [150]. Compared with the “gold standard” instrumentation, the proposed wearable electronic garment system features a favorable validity and agreement, which suggests the potential of using such a device to monitor strength and endurance exercises in vivo. Then, a microneedle array electrode-based wearable EMG system is proposed to detect the driver’s drowsiness when griping the steering wheel (Figure 14b) [151]. The results indicated that during driving, participants’ drowsiness levels increased while the activity of the muscles involved in the steering wheel grip decreased concurrently over time. The capturing of EEG is often limited by the very small signal amplitude of 50–100 μV, and the researchers mainly devote their efforts to developing high-precision electrodes to improve the quality of collected EEG signals. Shin et al. designed an earbud-like wireless EEG device (e-EEGd) that is composed of tattoo-like electrodes, connectors and a wireless EEG earbud. The tattoo-like electrodes and connectors show a good ability in decreasing direct noise from motion artifacts (2–4 Hz) and indirect noise (0–2 Hz). (Figure 14c) [152].

#### 4.1.4. Body Temperature

The development of body temperature sensors has been pursuing the features of wearability, high sensitivity, good accuracy, portability, an array with a large area and real-time monitoring capacity. The reported flexible temperature sensor mainly uses thermosensitive materials to transduce the temperature change into corresponding electrical signals. Generally, temperature sensing is achieved by thermal resistance, thermocouples and thermistors. Yu et al. presented a flexible thermal-resistance sensor made by sandwiching a PEDOT:PSS sensing film between two PDMS substrates [153]. Stable microcracks are engineered in the sensing by pre-stretching the sensor to bestow high sensitivity and linearity. The sensor successfully distinguishes the small rise (from 27.5 °C to 28.5 °C) of skin temperature before and after a 5-min running exercise and shows good consistency with commercialized IR thermograms (Figure 14d). Prof. Zhuangde Jiang’s team recently developed a thin thermocouple film with a combination of platinum and indium oxide (Figure 14e) [39]. Benefiting from the ultrathin characteristics, small heat capacity and fast response characteristics, the sensor can realize real-time monitoring of breath temperature.

### 4.2. Devices for Metabolism

Metabolism monitoring aims at the physical conditions of humans, and the involved parameters include the excretion volume of body fluids and the concentration of metabolites in these body fluids. Measuring the excretion volume mainly lies in the effective collection of fluids and high-accuracy volume characterization, which often requires help from microfluidics. The concentration of several biochemical metabolic markers, e.g., glucose, lactate and electrolytes (Na^+^, Ca^2+^, K^+^, Cl^−^), are evaluated by electrochemical sensors. Sweat, saliva, tears and interstitial fluid (ISF) have been used as samples, and sweat is the most common one. In the following part, the available devices for metabolism monitoring will be sequentially introduced according to the targeted samples. 

#### 4.2.1. Devices for Sweat Analysis

The first concern of sweat analysis in sports is sweat loss (SL). There are two types of human sweat under normal conditions, namely, sensible sweat in the form of liquid and insensible sweat in the form of vapor. Typically, the human sweat rate is in the range of 10–2000 g/(m^2^·h), where 90–2000 and 10–90 g/(m^2^·h) represent sensible and insensible sweat, respectively. Athletes often need measuring devices with a larger measurement range due to their prominent thermoregulatory sweating. Conventional sweat loss measuring devices (SLMD) often use a patch or capsule to collect and indicate the value by hygrometry and gravimetry [77]. Though many of these methods exhibit high reliability, the lack of real-time performance may lead to a lag in the evaluation. The recently emerging progress in wearable sweat loss measuring devices (W-SLMD) gives this field a booming development. In accordance with the used principle, the available devices can be categorized into hygrometer-based, absorbent-material-based and microfluidics-based ones [154]. 

Similar to conventional devices, hygrometer-based W-SLMD also integrates humidity sensors into the system, but the wearable and flexible characteristics are pursued [155]. Wearability is the main target for the research of W-SLMD. For example, Salvo et al. developed a wearable sensor for the real-time measurement of sweat rate in localized areas of the human body to monitor athletes’ hydration status during training and improve their performances [78]. Two commercial humidity and temperature sensor chips (SHT25) are inserted into a wristband to capture the SL signal. The sensor has a working range of up to 400 g/(m^2^·h), and the obtained results from thirteen football players prove that this sensor is comparable to the medical device (Dermalab) that is used as a gold standard. Sim et al. put a capacitive humidity sensor into a humidity chamber and integrated them with batteries and a thermopneumatic actuator into a watch type W-SLMD [80]. The proposed sensor has a sensitivity (capacitance rising rate) of 0.039 (pF/s)/(g/m^2^·h) and linearity of 97.9% in the human sweat rate range. These devices have competitive performance when compared with gold-standard medical devices, but the rigid components and large volumes still give the users extra uncomfortableness. 

**Figure 15:**
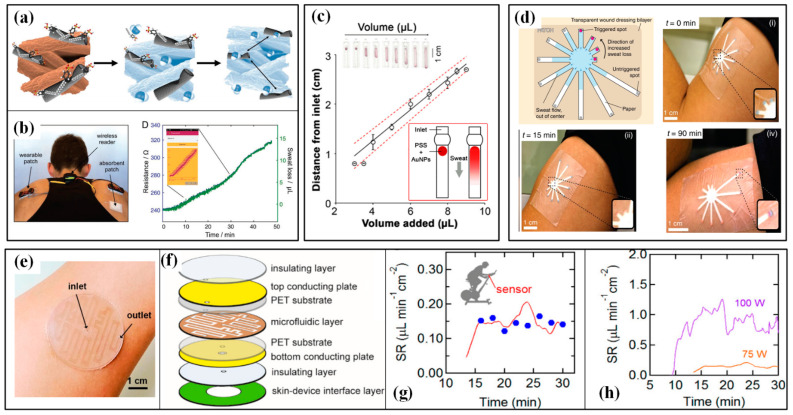
The wearable sweat loss sensors and their applications in sport monitoring: (**a**) the sweat-induced increment in the electrical resistance of CNT-doped conductive paper and (**b**) the monitoring of sweat loss during cycling sports [156]; (**c**) the simultaneous colorimetric detection of sweat volume [157]; (**d**) the mass-customizable dermal patch for calorimetrically quantifying sweat rate [158]; (**e**) the microfluidics-based capacitive sweat rate sensor, (**f**) its schematic diagram and obtained results (**g**) when comparing with Macroduct (blue dots) and under (**h**) different cycling power [162]. Reproduced with permissions from Wiley (2019) [156]. Reproduced with permissions from American Chemical Society (2021) [157]. Reproduced with permissions from American Chemical Society (2020) [162].

Different from the patch in the conventional gravimetry method, the absorbent-material-based W-SLMDs possess functional components to transduce the SL signal into readable signals in real-time. Paper, fabric, hydrogel and sponge have been utilized as absorbent materials to efficiently collect sweat. Electrical and colorimetric signals are the main forms of readable signals. Due to the hygroscopic expansion of cellulose, which increases the distance between each CNTs, the CNT-doped conductive paper will produce an increment in its electrical resistance when it is moisturized by sweat (Figure 15a) [156]. After a necessary calibration, this device can be used to monitor the SL during cycling sports (Figure 15b) [156]. The swelling of hydrogel under sweat also generates an obvious strain and changes the resistance of embedded strain-sensing fabric. Insensitive to body movement and interferences in daily life, this sensor features good reliability and completive performance compared with conventional gravimetric analysis [84]. When changing the flexible electronics into colorimetric materials, the SL value can be assessed by more readable color signals. The following two technical routes are often used: the former stores functional colorants in a pre-prepared reservoir and releases them upon sweat absorbance, and the SL is estimated by measuring the length of the stained substrate; the latter uses paths with different distances between the sweat absorber and colorimetric material, and the color change induced by the full saturation of each path indicates a certain sweat volume. Obviously, the first scheme can obtain a continuous and quantitative measurement of SL, but a careful gauge is needed (Figure 15c) [157]; the latter has a flaw in showing continuous values, but it can provide a direct and easy way to evaluate whether the SL reaches a pre-set value or not (Figure 15d) [158]. 

The microfluidics-based W-SLMDs refer to the devices that use microfluidics as the core component for collecting, transferring or storing sweat [12]. The SL value captured by microfluidics-based W-SLMD is also indicated by electrical or colorimetric signals, but the participation of winded microfluidics brings better flexibility, higher integration and smaller size/weight. For colorimetric dives, the functional dyes are arranged near the inlet or filled in the channels, and the length of the stained or color-changed channel is corresponding to the level of SL. As a pioneer, the group of Prof. Rogers has proposed a series of colorimetric microfluidics-based W-SLMDs [159,160,161]. The microchannels are usually made of flexible polymers, e.g., PDMS, polyurethane and superior geometries are proposed to render the devices with specific characteristics, such as waterproof and being resettable. As for the electrical W-SLMDs, microfluidics is often used as an intermediary to correlate the electrical signals to the level of SL. For example, Choi et al. proposed a microfluidics-based capacitive sweat rate sensor for continuous and real-time monitoring of sweat loss (Figure 15e) [162]. As illustrated in Figure 15f, the microfluidic layer is sandwiched between two conductive plates, forming a plane-parallel capacitor. During perspiration, sweat enters through the inlet hole and the microfluidic channel is progressively filled. Due to the significant difference in permittivity between empty (filled by air) and filled channels, the sensor will output a variation in its capacitance. The sensor can be used to monitor exercise-induced sweating under different intensities, and the results prove a favorable consistency with the Macroduct collection device (Figure 15g,h). 

Another core target of sweat analysis is obtaining the concentration of typical metabolites. As there is a correlation between the concentration of metabolites in sweat and blood plasma, non-invasive monitoring of sweat can be used to assess the metabolism of athletes. Electrolytes (e.g., Na^+^, Ca^2+^, K^+^, Cl^−^) and biomolecules (e.g., glucose, lactate and uric acid) have been tested by several wearable devices through electrochemical and colorimetric sensing principles. Potentiometry is an often-used method to measure the concentration of ions, which transduces the ion activity into an electrical potential by following the Nernst equation. Facing the concurrence of multiple ions, ion selectivity is a key feature for the sweat sensor to accurately represent the parameters of every kind of ion. Therefore, an ion-selective electrode (ISE) is often constructed by hybridizing an ionophore and conductive patterns. Each ionophore only responds to a certain ion, which is the core element for achieving high ion selectivity. The ISE acts as the working electrode and a reference electrode is required. Biomolecules are often measured by the amperometric method with the help of enzyme recognition elements. Similar to ion sensing, enzymes undertake the molecule selectivity task and act as the working electrode to catalyze a redox reaction to initiate an electron transfer process between the redox center of the enzyme and the working electrode. 

**Figure 16:**
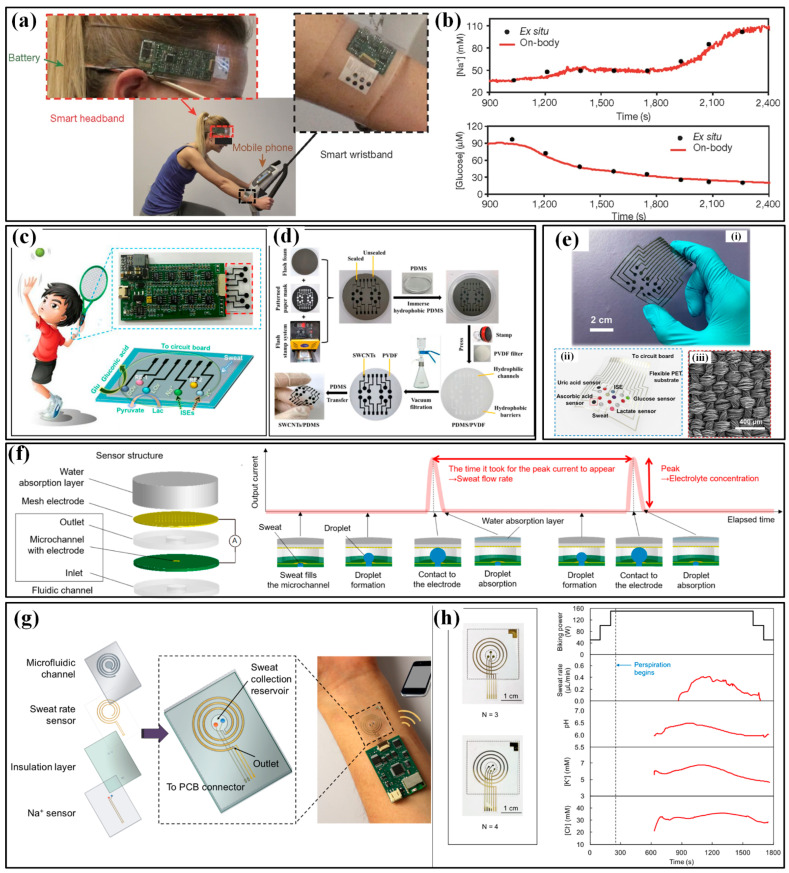
The wearable sweat metabolite sensors and their applications in sport monitoring: (**a**) the wristband and headband to monitor the perspiration during stationary leg cycling and (**b**) the obtained results [35]; (**c**) the sensor array for simultaneously detecting glucose, lactate, Na^+^ and K^+^ in sweat and (**d**) its fabrication based on vacuum filtration–transfer printing [34]; (**e**) the photo (i), schematic (ii) and electrode made of carbonized silk (iii) of the sensor that can detect six biomarkers is achieved by carbonizing a silk fabric textile [168]; (**f**) the microfluidic sensor for simultaneously monitoring the SL and electrolyte concentration [81]; (**g**) the flexible microfluidic sweat sensing patch for real-time electrochemical sensing and sweat rate analysis and (**h**) the capacity of modifying the number of electrodes [170]. Reproduced with permissions from American Chemical Society (2022) [34]. Reproduced with permissions from Springer Nature (2016) [35]. Reproduced with permissions from American Chemical Society (2018) [170].

Obviously, it is the core task for ion and biomolecule sensors to find the proper material for the working electrode to achieve high selectivity. Meanwhile, benefiting from the high selectivity, the capacity of wearable sweat sensors has gradually developed from focusing on a single analyte (e.g., pH [163], Na^+^ [164], Cl^−^ [165], NH4^+^ [166] or glucose [167]) to simultaneously measuring multiple targets. Wei Gao’s group has reported a fully integrated wearable sensor array for simultaneously and selectively analyzing four different parameters in sweat, including glucose, lactate, Na^+^ and K^+^ (Figure 16a) [35]. The sensor array can be packaged into a wristband or headband to monitor perspiration during stationary leg cycling. The accuracy of in situ measurements is verified through the comparison of on-body sensor readings from the forehead with ex situ (off-body) measurements from collected sweat samples (Figure 16b). Similarly, Hao et al. also reported a sensor array for simultaneously detecting glucose, lactate, Na^+^ and K^+^ in sweat and the captured values during fitness exercises were verified by ex situ devices (Figure 16c) [34]. The flexible CNT electrode array is fabricated by a simple, low-cost and eco-friendly vacuum filtration–transfer printing method, and the strategy can be easily expanded to the economical manufacturing of other flexible electronic devices (Figure 16d). The detection of six biomarkers is achieved by carbonizing a silk fabric textile (Figure 16e) [168]. The highly conductive textile features a hierarchical woven, porous structure and can be directly used or combined with other compounds to serve as the working electrode. Six biomarkers, namely, glucose, lactate, ascorbic acid, uric acid, Na^+^ and K^+^, are simultaneously detected with high sensitivity, good selectivity and long-term stability. 

Further integration is realized by combining the SL sensor and biomarker sensors [13,169]. Hashimoto et al. used a microfluidic sensor to simultaneously monitor the SL and electrolyte concentration [81]. As shown in Figure 16f, the SL is assessed from the time for the droplet to appear and the droplet volume, and the concentration in each droplet is indicated by the peak value in the obtained current. Similarly, a flexible microfluidic sweat sensing patch for real-time electrochemical sensing and sweat rate analysis is presented [170]. As illustrated in Figure 16g, this device contains the following four layers: a spiral-patterned microfluidic component for transporting sweat, a pair of parallel Au electrodes for electrical impedance-based sweat rate sensing, a parylene-C layer for insulation and an ion-selective layer for measuring electrolyte concentration. By properly modifying the number of electrodes (N = 3 or 4), two or three sweat analytes are detected, in addition to sweat rate (Figure 16h).

#### 4.2.2. Devices for Saliva, Tear and ISF Analysis

**Figure 17:**
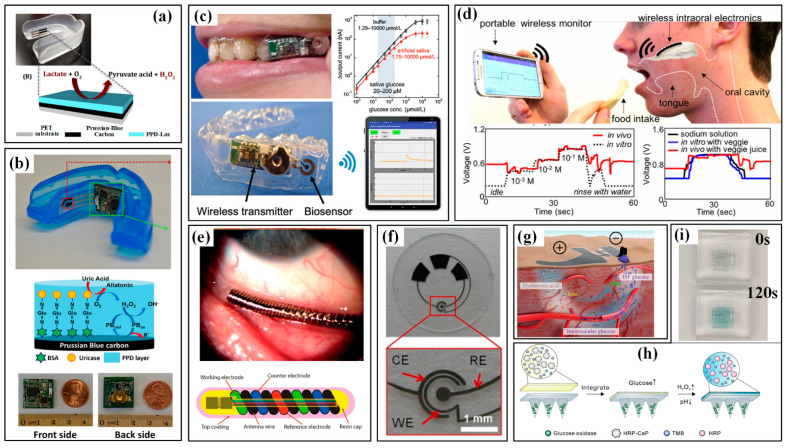
The wearable sensors for saliva, tear and ISF analysis: (**a**) the mouthguard for salivary lactate monitoring [171]; (**b**) the photo (up), mechanism (middle) and circuit photo (down) of the mouthguard for salivary uric acid monitoring [172]; (**c**) the mouthguard for salivary glucose monitoring [173]; (d) the intraoral sensors for real-time quantification of sodium intake [174]; (**e**) the sensor for monitoring the tear glucose [175]; (**f**) soft contact lens for monitoring the tear glucose [177]; (**g**) illustration for monitoring the ISF glucose based on reverse iontophoresis [179]; (**h**) the microneedles array for monitoring the ISF glucose and (**i**) its colorimetric results [180]. Reproduced with permissions from The Royal Society of Chemistry (2014) [171]. Reproduced with permissions from Elsevier (2015) [172]. Reproduced with permissions from American Chemical Society (2020) [173]. Reproduced with permissions from The National Academy of Sciences (2018) [174]. Reproduced with permissions from Elsevier (2020) [180].

The saliva-based physiological monitoring, namely, salivaomics, is conducted by integrating the devices into mouthguards (MG), pacifiers and teeth. The main targets of salivaomics are the biomolecules related to human metabolisms, such as lactate, uric acid and glucose. For example, a 3-electrode system with a PPD-LOx enzyme working electrode is attached to the inner side of an MG for salivary lactate monitoring (Figure 17a) [171]. Similarly, a sensing system with Prussian-blue-graphite electrodes is used to detect the uric acid, and the obtained signals can be wirelessly transmitted through BLE by the integrated amperometric circuit board (Figure 17b) [172]. Then, the monitoring of saliva glucose is realized by Arakawa et al., and the signals are also transmitted with the help of a BLE-based circuit (Figure 17c) [173]. Meanwhile, the intraoral sensors can also be used to monitor food intake, which is also an important concern for athletes in daily life. For example, Lee et al. reported intraoral hybrid electronics for the real-time quantification of sodium intake (Figure 17d) [174]. The sensing system has a multi-layered structure, containing a microstructured ion-selective sodium sensor, signal filtering/amplification, Bluetooth low-energy wireless telemetry, an antenna and a miniaturized microcoin battery. When mounted in the mouth by a custom-fit retainer, the sensor shows a high sensitivity to sodium intake. 

The wearable tear sampling techniques can be implemented by putting a tear sensor into the eye or integrating it with a soft contact lens (SCL). The former requires a minimized size to maintain comfortableness. For example, a tear sensor made of micron-sized Pt/Ir coils, enzyme and UV curable glue is put into the lower eyelid to wirelessly monitor the tear glucose (Figure 17e) [175]. The latter SCL-based scheme is more popular. The group headed by Dr. Parviz has developed a serial of SCL-based tear sensors for monitoring lactate and glucose [176]. As shown in Figure 17f [177], the two/three-electrode biomarker sensor is integrated into the SLC to assess lactate or glucose, and then the coils are used to achieve wireless communication and receive the microwave energy to power the system.

ISF steadily exists in the dermis, salivary glands and sweat glands, making it a favorable candidate for continuous physiological monitoring. The concentration of ISF glucose, having a good consistency with that of plasma, has been measured by different technical routes. In the first route, reverse iontophoresis is conducted by driving the biomolecules in the ISF towards the skin surface with a mild electric current [178]. Chen et al. used a paper battery to generate subcutaneous electrochemical twin channels. Along with the hyaluronic acid penetration into ISF (anode channel), the glucose also reverses iontophoresis to the skin surface (cathode channel) (Figure 17g) [179]. Though many efforts have been devoted to decreasing the loaded current, skin discomfort and pain still exist. Therefore, another route based on microneedles (MNs) has been developed. For example, a PVA-based double-layer microneedle patch is prepared to achieve both in situ dermal sample collection and instant color display [180]. Glucose oxidase is packaged in the MNs to selectively convert glucose into gluconic acid and H_2_O_2_. The lowered local pH and the presence of hydrogen peroxide cause a color change in the immobilized upper layer (Figure 17h). This colorimetric sensor successfully achieves minimally invasive extraction of the interstitial fluid from mice and converts glucose level to a visible color change promptly (Figure 17i).

## 5. Multifunctional Flexible Sensors for Sports

As illustrated in the above sections, the great progress in flexible, wearable sensors endows sports with plenty of tools to monitor the exercise process, moving actions and athlete status. Several kinematical and physiological parameters have been captured by high-performance wearable devices. Moreover, the power of the monitoring system is still being driven to further advancement. In order to reach the ambitious goal of “All-in-One”, the multi-functionality of wearable monitoring systems becomes a core pursuit in this domain to acquire diverse signals of motion, vital signs and metabolism with only a single device. The multi-functionality mainly deals with the following two issues: i) introducing more sensing capacities into the system and ii) constructing more auxiliary components. The available ways for integrating these different elements include building the whole system with inherent flexible materials and flexing the whole system by combining the minimized IC chips with flexible substrates. 

**Figure 18:**
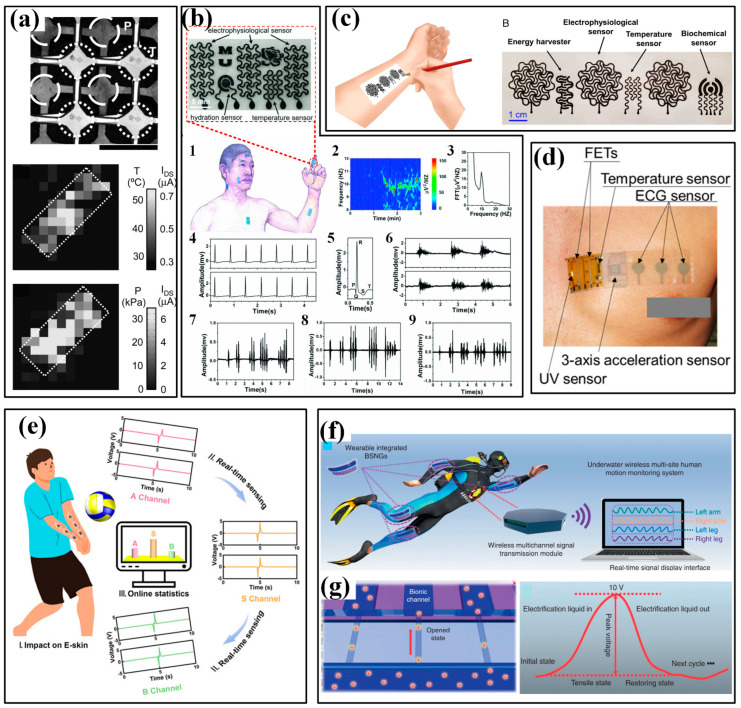
The wearable multifunctional sensors for sports: (**a**) the wearable pressure-temperature sensor and obtained distribution maps [181]; (**b**) the wearable device containing electrophysiological sensors, skin hydration sensors, temperature sensors/joule-heating elements and the obtained results of Alpha rhythm (2),center of Alpha rhythm (3), ECG signals (4), refined P,Q,T waves of ECG (5), EMG signals from the forearm (6) and EMG signals when the volunteer clenched the jaw (7), nodded the head (8), and bent a finger (9) [182]; (**c**) the pencil–paper on-skin multifunctional electronics [184]; (**d**) the multifunctional sensor for three-axis acceleration, ECG, temperature and UV [185]; (**e**) the TENG-based electronic skin to monitor the reception pressure in volleyball [188]; (**f**) the stretchable nanogenerator for underwater sensing and energy harvesting and (**g**) its working mechanism [189]. Reproduced with permissions from The National Academy of Sciences (2005) [181]. Reproduced with permissions from Wiley (2018) [182]. Reproduced with permissions from Wiley (2018) [182]. Reproduced with permissions from The National Academy of Sciences (2020) [184]. Reproduced with permissions from American Chemical Society (2021) [188].

The introduction of different sensing elements into a single patch is of great importance to simultaneously monitor multiple parameters during sports. The main challenge in multifunctional flexible sensors is the possible interference between multiple obtained signals, which may be subducted by proper sensor configuration and decoupling of sensing mechanisms. In the last two decades, many researchers have devoted great effort to blending different kinds of sensors into one flexible system. For example, a temperature sensor has been integrated with a pressure sensor to synchronously map the pressure and temperature distributions, and the excellent isolation of each sensor leads to more accurate sensing (Figure 18a) [181]. Moreover, the temperature sensor can be an important reliance for compensating the temperature influence in several electrochemical sensors. As for the sweat sensor in [35], a temperature sensor is also integrated into the device to monitor the skin temperature and to eliminate the influence of temperature variation in the readings of the chemical sensors through the built-in signal processor. Then, the variety of integrated devices is increasingly expanded. Gao et al. produced a forehead EEG-sweat rate multifunction sensor by packaging an ST20 humidity-sensitive capacitance and a conductive fabric electrode [155]. A microporous film of PDMS above the fabric electrode provides a path for the generated sweat. The test results prove that the EEG electrode has a quite small contact impedance and similar performance compared with the conventional Ag/AgCl wet electrode, and that the measured sweat rate is also very close to the average sweat rate from the weighing method. Furthermore, with the advances in patterning techniques, more and more sensing elements are arrayed on one layer. Sun et al. patterned electrophysiological sensors, hydration sensors and temperature sensors/joule-heating elements on the surface of a silicone elastomer sponge with the help of a CO_2_ laser (Figure 18b) [182]. Skin hydration is indicated by the measured skin impedance and temperature is evaluated by measuring the resistance variation of the sensing element. Simultaneously, the laser-induced graphene is conductive and is used as electrodes for capturing EEG, ECG and EMG signals. Similarly, Gong et al. reported a single-material multifunctional sensor through a local-cracking technique [183]. Localized cracks with tunable sizes, shapes, and orientations are utilized to form strain/pressure sensors, anisotropic orientation-specific sensors, strain-insensitive stretchable interconnects, temperature sensors, glucose sensors and lactate sensors without the need for soldering or gluing, which enables the convenient monitoring of athlete’s body health status, such as sweat composition detection, motion monitoring and heat regulation. More recently, a very interesting work was reported on the pencil–paper on-skin electronics (Figure 18c) [184]. By using widely accessible pencils and office paper as tools, a variety of cost-effective and disposable devices are produced, ranging from biophysical sensors and sweat biochemical sensors to thermal stimulators, humidity energy harvesters, transdermal drug-delivery systems and antenna circuits. Some efforts are also devoted to developing multifunctional sensors for simultaneously monitoring kinematical to physiological signals. A representative device is a proof-of-concept, multifunctional flexible device reported by Yamamoto et al. As shown in Figure 18d [185], the realized devices included a printed three-axis acceleration sensor for motion detection, a group of CNT electrodes for ECG sensor, a CNT temperature sensor and an environmental ultraviolet sensor, and flexible field-effect transistors for the switching of sensors. The device is attached directly to the skin of the chest and the motion, skin temperature, ECG and UV exposure are successfully monitored. 

Some other works integrate more auxiliary components into a flexible substrate to enrich the capacities of wearable devices. Commonly, the hybrid integration of flexible PCB (FPCB) is realized to guarantee wearability. Firstly, the inherent flexible electronics, by now, cannot process captured signals as well as conventional ICs. Therefore, many reported works realize their wearable system by combining the prepared flexible sensors and the FPCBs with IC chips. For example, some of the abovementioned sweat sensing systems, shown in Figure 16a,c, use many IC chips to transduce the signals, process the values and transmit the results. Secondly, the properties of some flexible sensors cannot accurately measure the target parameters. The accelerometer is a kind of device that suffers from this problem. Though some flexible prototypes have been reported, their sensitivity, accuracy and linearity are all far away from the commercial MEMS accelerometers. So, the MEMS chips are also involved in the hybrid FPCBs. For instance, Zhao et al. reported a hybrid FPCB for simultaneously measuring ECG and body motions that integrated a MEMS accelerometer (ADXL345), an ECG chip (AD8232), an MCU chip (CC2640R2F), a voltage regulator (TPS61070) and a button battery (CR1220) [186]. The collected ECG and acceleration data are wirelessly transmitted and displayed in real-time on a mobile phone application through Bluetooth communication. Lastly, the power sources for wearable systems should also be considered. Conventional power sources and energy storages are usually bulky with circuital connections and demand frequent charging and replacement, which strongly restricts the practical applications of wearable devices in sports. However, external stimulations are not only triggering signals for sensing but also an important driver for electric power generation. Developing self-powered systems is a promising alternative strategy to solve the issue of energy supply. Flexible triboelectric nanogenerators (TENGs), firstly reported by Prof. Zhonglin Wang [187], convert the mechanical energy in body motions into electrical energy, which has been a very emerging breakthrough for self-powered wearable monitoring systems in sports. Shi et al. developed an all-fiber TENG-based electronic skin to monitor the reception pressure on arms in volleyball (Figure 18e) [188]. The two 2 × 3 integrated E-skin arrays are worn on both arms, and the waveform and amplitude of their output voltages are utilized to do the statistical analyses. The obtained motion sensing, position monitoring and distribution statistics are helpful to athletes and coaches in training and formulating competition strategies. By mimicking the structure of ion channels on the cytomembrane of electrocyte in an electric eel, Prof. Wang’s team proposed a stretchable nanogenerator for underwater sensing and energy harvesting (Figure 18f) [189]. Combining the effects of triboelectrification caused by flowing liquid and principles of an electrostatic induction, the bionic stretchable nanogenerator can harvest mechanical energy from human motion underwater and output an open-circuit voltage of over 10 V (Figure 18g). A wireless system for monitoring body motions underwater is constructed, and the motion signals of different parts of the human body are recorded when the volunteer swims in different styles. Meanwhile, some other flexible energy sources are being researched, and the available schemes include photovoltaic solar cells [190], biofuel cells that capture energy from body fluids [191] and thermoelectric generators [192].

## 6. Challenges and Prospects

The past decade has witnessed remarkable advances in flexible, wearable sensor devices, from working mechanisms, functional materials and fabrications to imaginative applications. Sports monitoring also benefits a lot from this booming research field, and many of the body signals during exercise, from kinematics to physiologies, have been detected by wearable sensing devices. However, the flexible sensors in sports monitoring with high sensitivity and stability are still in their infancy. Many challenges are still existing on the development road, such as the maturity, long-term reliability under various conditions, long-distance transmission of obtained signals, high-efficiency signal processing methods, wearable display to show the results in real-time and the possible adverse effects on athletes. In the following parts, these challenges are discussed, accompanied by the prospects of possible solutions. 

Firstly, better maturity calls for more distinguish works that apply the wearable sensors in real sport activities. Currently, most of the reported devices are validated at the research stage, and the monitoring results are obtained from the simulated environments in the laboratory. The lack of actual combat assessment undermines the viability of monitoring results and then influences the adoption by athletes and coaches. The experimental results from actual combat should be compared with the “gold-standard” equipment to verify the confidence level of these newly developed wearable systems. For example, the motion recording data from wearable systems can be verified by the multiple camera-based video motion capture system, and the ECG/EMG signals can be a good challenger for the ones from conventional wet electrodes. Though basic verifications have been conducted in several works, but their depth, breadth and duration are far away from the requirements of commercial products. Therefore, the better maturity of wearable sensors should be achieved through more systematic, comprehensive practical verification, especially focusing on the comparisons between the results and the widely accepted gold-standard approaches. 

The long-term stability and reliability under the sport conditions are another concern. The surrounding environments on sport fields is complex and varied. The temperature and humidity may induce a great deviation in the measuring characteristics of the sensor. Then, the mechanical impact and external abrasion during sporting may decrease the device’s functions and the whole device may even be damaged. It has been proved that many piezoresistive, capacitive and electrochemical flexible sensors have a great temperature coefficient in their sensing features, which will bring out significant deviations in the measured results without necessary compensations [193,194]. Temperature sensors have been integrated into many sensing systems to get rid of the dispreferred effects when a subsequent compensation algorithm is added. Humidity is also a critical parameter in this field, and its influence can also be subducted by compensation methods. Moreover, the high performance of the device in swimming and hyperhidrosis should be achieved by the excellent water resistance of devices, and the practical methods include introducing hydrophobic chemical groups or generating surface nanostructures [195,196,197]. The mechanical impact or abrasion can exfoliate the functional layers of sensors and even break the whole sensor structure. There are the following two feasible paths to solve this issue: improving the packaging strategy to promote the device viability and pursuing self-healing ability for the devices. 

**Figure 19:**
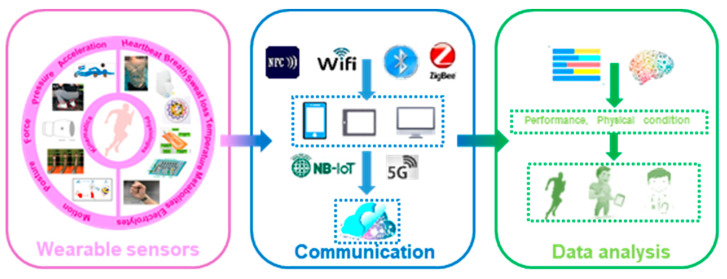
The capture, transmission and processing/analyzing of data in sports.

The transmission and processing of signals are also challenging. Wire transmission of obtained signals has been abandoned in many sports due to its limited distance and extra burden for athletes. However, the common wireless techniques (e.g., NFC, BLE and Wi-Fi) often suffer from the operating distance when they appear on the sports fields. A more practical scheme is constructing a relay station, which captures the signals through near-distance communication and transmits them to the cloud by long-haul communication (e.g., NB-IoT, LTE and 5G). As shown in Figure 19, the kinematical and physiological signals are sensed by the wearable systems and transmitted to the cloud by the joint communication channel. Then, the data can be processed and analyzed to produce a training or competing report for the athletes or coaches. As for the signal process, the massive data, multiple parameters and diverse analyzing targets make this work a task that requires huge efforts and calculations. Recently, several intelligent methods are proposed to classify and recognize the motions during sports. The implemented algorithms include artificial neural network (ANN), convolutional neural network (CNN), long short-term memory (LSTM) network, k-nearest neighbor (KNN), decision tree and support vector machine (SVM), etc. [198,199,200,201,202]. A large number of sports have been treated in the classifying work, such as golf, racquet sports, swimming, running, race-walking and so on [27]. Few works aim at evaluating the physical condition of athletes, and fewer papers focus on assessing athleticism through obtained monitoring data [201]. In the future, the continuous advances in artificial intelligence may provide more powerful tools for coaches and athletes to accurately assess competing performance and physical conditions.

The wearing comfort and fitness are important for professionals. The flexible devices have minimized the wearing discomfort by greatly improving skin compliance, reducing the device weight/size and introducing breathability. However, the safety of utilized materials, such as biocompatibility, non-toxic, nonirritant and unrelated with excitants, still needs further investigation, which may be addressed by the green technologies in material science and fabrication process. 

Last and probably the hardest, is the realization of intrinsically flexible, fully functional wearable monitoring systems for sports, in which the signal collector, transmitter, processor and displayer are supposed to be flexible and self-powered. However, the energy density of flexible power suppliers is still limited; the calculating functions of flexible electronics are much weaker than conventional ICs; the wearable displayers are only preliminarily verified. These issues require unremitting researches in basic mechanisms, material science and manufacturing techniques in the future.

## 7. Conclusions

In the past few years, tremendous progress has been made in flexible and wearable sensors, and extensive applications have been realized in health monitoring, human–machine interaction, the Internet of Things, artificial intelligence and other fields. In this review, we summarize the current developments in utilizing flexible and wearable sensors in monitoring the kinematical and physiological signals of sports. Some typical and representative indicators for evaluating the performance and physical condition of athletes, including motions of the body, force/pressure acting on the body, vital signs and metabolizing parameters, are comprehensively illustrated. The available wearable devices and their applications towards the related indicators, including strain sensor, pressure/force sensor, IMU, electrophysiological electrode, sweat loss sensor and electrochemical sensor, are emphatically presented. Despite the great progress made in this burgeoning topic, challenges still exist for practical sports applications. The possible solutions rely on future researches in basic mechanisms, material science and manufacturing techniques.

## Data Availability

Not applicable.
